# Complete genome sequencing and comparative genomic analyses of *Bacillus* sp. S3, a novel hyper Sb(III)-oxidizing bacterium

**DOI:** 10.1186/s12866-020-01737-3

**Published:** 2020-05-01

**Authors:** Jiaokun Li, Tianyuan Gu, Liangzhi Li, Xueling Wu, Li Shen, Runlan Yu, Yuandong Liu, Guanzhou Qiu, Weimin Zeng

**Affiliations:** 1 School of Minerals Processing and Bioengineering, Central South University, Changsha, 410083 China; 2 Key Laboratory of Biometallurgy, Ministry of Education, Central South University, Changsha, 410083 China

**Keywords:** *Bacillus* sp. S3, Sb(III)-resistance, Genome sequencing, Comparative genome, Heavy metal (loid)s

## Abstract

**Background:**

Antimonite [Sb(III)]-oxidizing bacterium has great potential in the environmental bioremediation of Sb-polluted sites. *Bacillus* sp. S3 that was previously isolated from antimony-contaminated soil displayed high Sb(III) resistance and Sb(III) oxidation efficiency. However, the genomic information and evolutionary feature of *Bacillus* sp. S3 are very scarce.

**Results:**

Here, we identified a 5,436,472 bp chromosome with 40.30% GC content and a 241,339 bp plasmid with 36.74% GC content in the complete genome of *Bacillus* sp. S3. Genomic annotation showed that *Bacillus* sp. S3 contained a key *aioB* gene potentially encoding As (III)/Sb(III) oxidase, which was not shared with other *Bacillus* strains. Furthermore, a wide variety of genes associated with Sb(III) and other heavy metal (loid) s were also ascertained in *Bacillus* sp. S3, reflecting its adaptive advantage for growth in the harsh eco-environment. Based on the analysis of phylogenetic relationship and the average nucleotide identities (ANI), *Bacillus* sp. S3 was proved to a novel species within the *Bacillus* genus. The majority of mobile genetic elements (MGEs) mainly distributed on chromosomes within the *Bacillus* genus. Pan-genome analysis showed that the 45 genomes contained 554 core genes and many unique genes were dissected in analyzed genomes. Whole genomic alignment showed that *Bacillus* genus underwent frequently large-scale evolutionary events. In addition, the origin and evolution analysis of Sb(III)-resistance genes revealed the evolutionary relationships and horizontal gene transfer (HGT) events among the *Bacillus* genus. The assessment of functionality of heavy metal (loid) s resistance genes emphasized its indispensable role in the harsh eco-environment of *Bacillus* genus. Real-time quantitative PCR (RT-qPCR) analysis indicated that Sb(III)-related genes were all induced under the Sb(III) stress, while *arsC* gene was down-regulated.

**Conclusions:**

The results in this study shed light on the molecular mechanisms of *Bacillus* sp. S3 coping with Sb(III), extended our understanding on the evolutionary relationships between *Bacillus* sp. S3 and other closely related species, and further enriched the Sb(III) resistance genetic data sources.

## Background

The hazardous heavy metal (loid) s, such as antimony (Sb), arsenic (As), cadmium (Cd), chromium (Cr) and lead (Pb) exert a serious threat to the natural environments and human health in many parts of the world [1–3]. In recent decades, natural biogeochemical cycle and anthropogenic activities including mining activities, rapid urbanization, and industrialization have contributed to elevated levels of heavy metal (loid) s in soils [4, 5]. Conventional remediation technologies have been developed to remove heavy metal (loid) s from contaminated surroundings, such as ion exchange, membrane separation, coagulation/flocculation, electrochemical methods, extraction and adsorption [6, 7]. However, most of these physiochemical remediation methods are not suitable for large-scale applications because of their high cost, generation of secondary pollution and unsustainable nature [8]. By contrast, microorganism-mediated bioremediation is an alternative promising technology due to its low-cost and environmentally friendly advantages [9]. Microorganisms are able to alleviate the toxicity of heavy metal (loid) s using various resistance strategies, such as extracellular precipitation, intracellular sequestration, enzymatic transformation and oxido-reduction of toxic metal ions [8, 10].

Antimony (Sb) is a toxic metalloid in group 15 of the periodic table of elements and excessive Sb can cause harmful damages to the human health [1, 4]. Sb and its compounds therefore have been listed as priority pollutants by the United States Environmental Protection Agency (USEPA) and the European Union (EU) [7, 11]. The maximum acceptable concentration of Sb in drinking water has been set at 6 μg/L by the World Health Organization (WHO) [12]. Because it’s widely used in flame retardants, Pb-Sb alloys, brake linings, and catalysts for polyethylene glycol terephthalate [4, 13], a sharply increasing release of Sb in the environments occurs during the past decades [1]. The main Sb species include antimonite [(Sb(III)] and antimonate [Sb(V)] in soil and water systems, which can be interconverted via biogeochemical processes. Sb(V) is more stable in aerobic environments than Sb(III), and Sb(III) is more toxic than Sb(V) due to its high affinity with thiol-containing proteins [14]. Thus, microbial Sb(III) oxidation that can transform the toxic Sb(III) to the less toxic Sb(V) has a significant value for the environmental Sb bioremediation [15].

In recently, more than 60 Sb(III)-oxidizing bacteria, including *Shinella* sp. strain NLS1, *Ensifer* sp. strain NLS4, *Acinetobacter* sp. JL7, *Comamonas* sp. JL25, *Comamonas* sp. JL40, *Comamonas* sp. S44, *Stenotrophomonas* sp. JL9, and *Bosea*sp. AS-1 have been isolated from different Sb-contaminated sites [7, 13, 14, 16]. *Bacillus* sp. S3 is a new hyper antimony-oxidizing bacterium, which has been previously isolated from contaminated mine soils [17]. Our previous study confirmed that it exhibited high Sb(III) oxidation efficiency (50 μM·d^− 1^) and Sb(III) resistance (5.5 mM) [17]. Meanwhile, this bacterial strain has been proved to occupy the ability to cope with multiple heavy metal (loid) s through various adaptive strategies [18]. Although increasing numbers of studies have focused on microbial Sb oxidation, the mechanisms of Sb transformation in *Bacillus* sp. S3 have not been well explored so far. Meanwhile, the lack of data on its genome sequence has restricted molecular studies and practical applications.

Furthermore, it is generally noted that *Bacillus* is a large genus of the gram-positive, heterotrophic, endospore-forming bacteria and belongs to Class: Bacilli, Phylum: Firmicutes [19]. Members of the *Bacillus* genus provide a model system for the study of metal ions and exhibit broad resistance to heavy metals [20]. With the extending of the third-generation sequencing platform PacBio RSII, large numbers of the *Bacillus* strains (more than 4551) have been genomic sequenced. In contrast, there are few literatures on Sb(III) oxidation and resistance mechanisms in *Bacillus* genus. The arsenite oxidase AioBA responsible for As (III) oxidation in *Agrobacterium tumefaciens* 5A was reported to also function as a Sb(III) oxidase [12]. Moreover, arsenite oxidase AioAB is composed of a large (AioA) and a small (AioB) subunit [21]. A novel Sb(III) oxidase AnoA was discovered to catalyze Sb(III) oxidation in *Agrobacterium tumefaciens* GW4 with NADP^+^ as the co-factor [22], the cellular H_2_O_2_ catalyzed bacterial Sb(III) oxidation as an abiotic oxidant [23]. Although these studies provide great advance, there has never been a comprehensive research focused on Sb(III) oxidation in terms of whole genome and comparative genomics. To further understand the molecular details of *Bacillus* sp. S3 against Sb(III), it is essential to determine the genomic information of Sb(III)-resistance strains. Here, genomes sequence and the comparative genomic analyses were applied to study the Sb resistance mechanism and evolutionary relationship of *Bacillus* sp. S3.

## Results

### Morphological characterization of *Bacillus* sp. S3

**Fig. 1:**
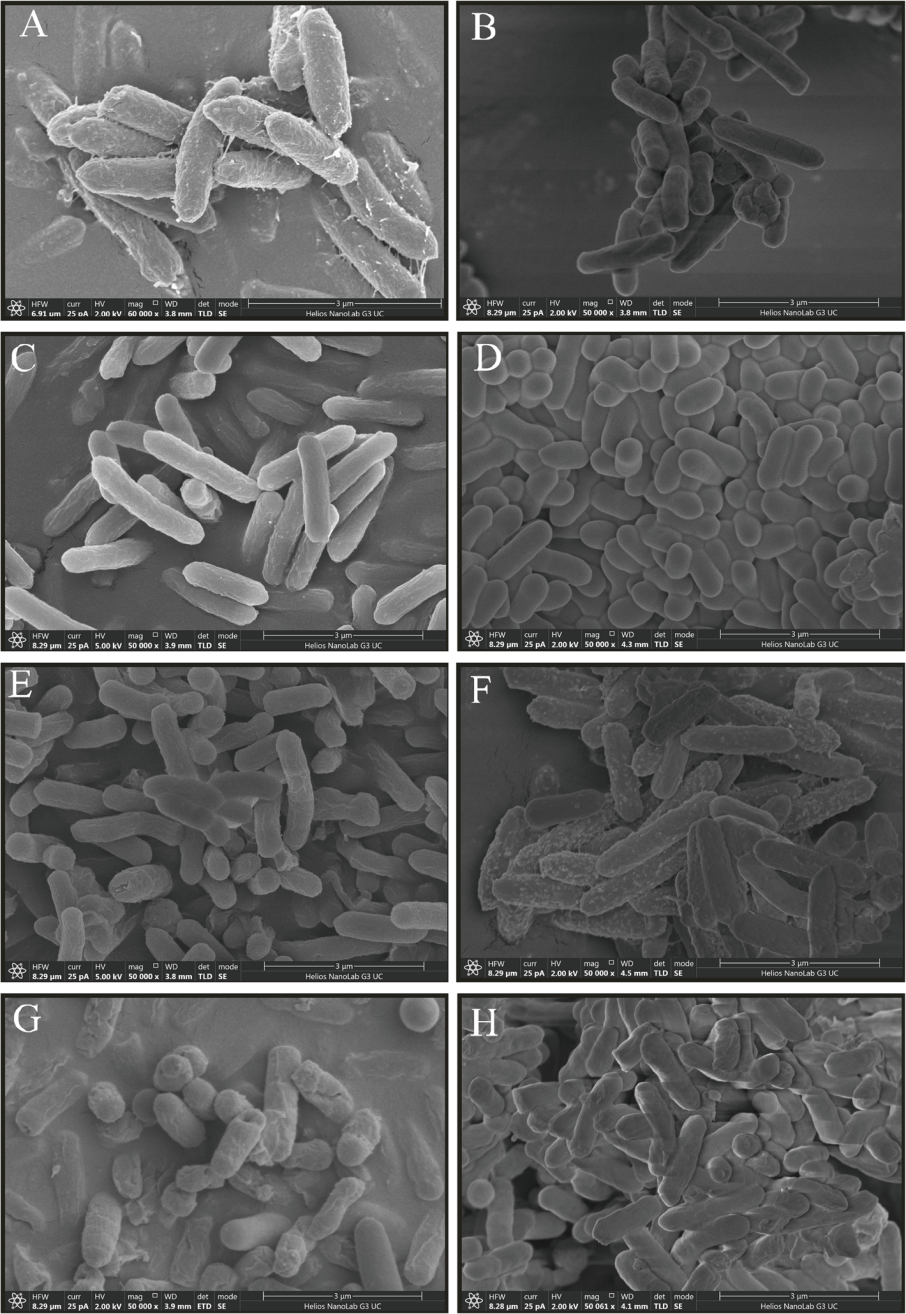
Scanning electron microscope (SEM) micrograph of *Bacillus* sp. S3 before and after the different heavy metal ions exposure: (**a**) CK; (**b**) Sb(III); (**c**) As (III); (**d**) Cd (II); (**e**) Cr (VI); (**f**) Pb (II); (**g**) Cu (II); (**h**) Zn (II)

As shown in Fig. 1a, the SEM images of *Bacillus* sp. S3 intuitively showed that the cell walls were enveloped by filaments, possibly due to the presence of extracellular polymeric substances. Intriguingly, after cultivation of *Bacillus* sp. S3 for exponential phase under the Sb(III) stress, the cell surface became smoother than control (Fig. 1b). Meanwhile, smaller cell size, lesser wrinkled cell wall and the occurrence of intracellular dissolution were visible in present of other heavy metal (loid) s (Fig. 1c-h). As shown in Figure S1A, no physical Sb(III) adsorption was detected on *Bacillus* sp. S3 cell surfaces by EDS analysis, which was similar to other studies [9]. When the initial concentration was 1 mM Pb (II), the peak value of EDS was significant from that of the control group and other treatment groups (Figure S1E).

### General genome feature of *Bacillu*s sp. S3

**Fig. 2:**
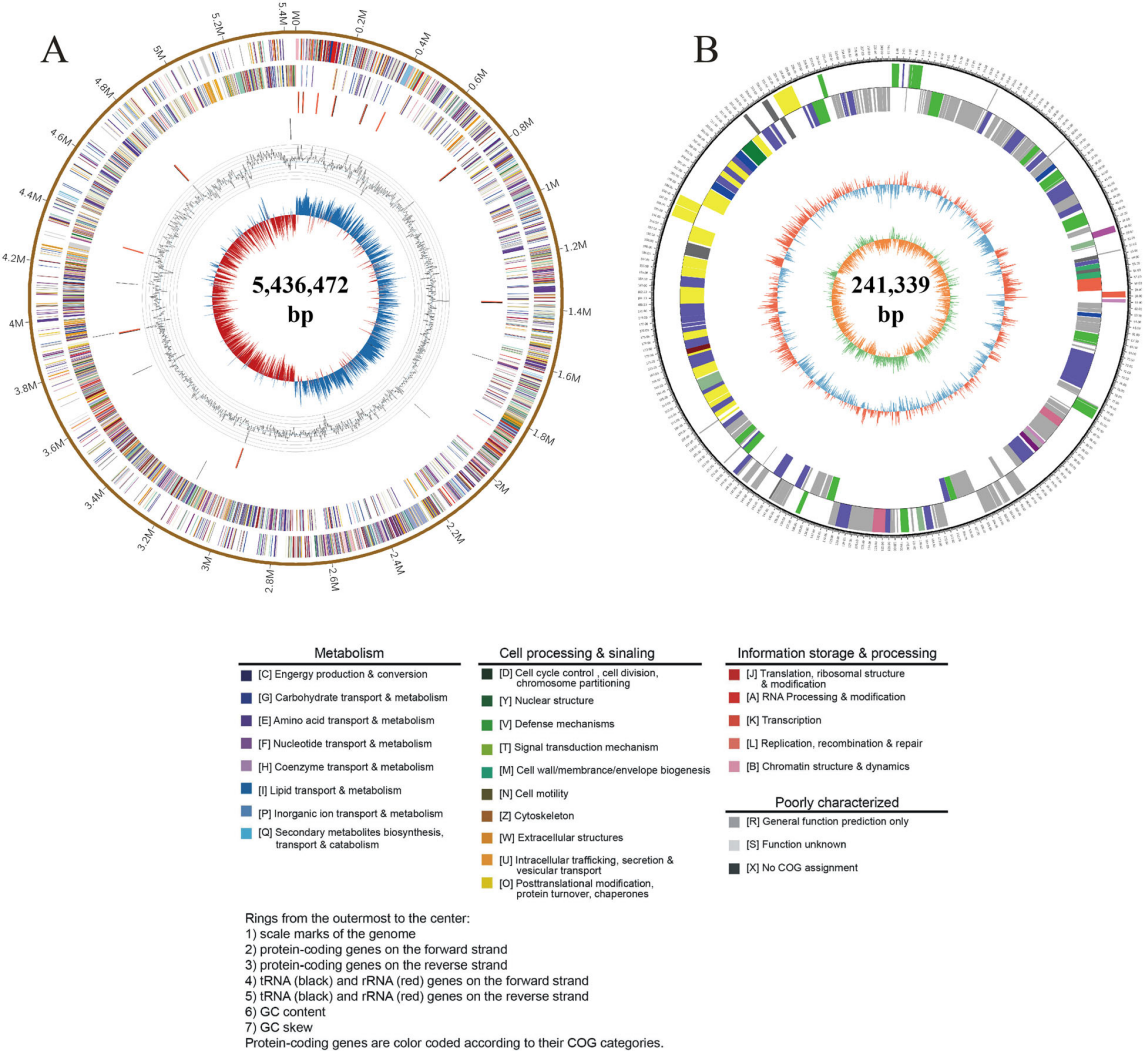
Circular genome maps of *Bacillus* sp. S3 chromosome (**a**) and plasmid (**b**). From the outer to the inner circle: (1) scale marks of genomes; (2) assigned COG classes of protein-coding genes (CDSs) on the forward strand as indicated by relevant colors; (3) forward strand CDSs; (4) tRNA (black) and rRNA (red) genes on the forward strand; (5) tRNA (black) and rRNA (red) genes on the reversed strand; (6) GC content (swell outward/inward indicates higher/lower G + C compared with the average G + C content); (7) GC skew (cyan/red indicate positive/negative values)

The general genomic features of *Bacillus* sp. S3 were summarized in Table 1 and Fig. 2. The whole genome of *Bacillus* sp. S3 contained one single circular chromosome of 5,436,472 bp with 40.30% GC content and one plasmid of 241,339 bp with 36.74% GC content (Fig. 2a, b). The whole genome harbored 5131 protein-coding sequences covering 85.35% of the genome with the average length of 904 bp, as well as 36 rRNAs and 104 tRNAs (Table 1). In addition, the genomic features of *Bacillus* sp. S3 and the other 44 closely related strains were summarized in Table 2. The average chromosome length of the 45 *Bacillus* genomes was 4.99 Mb with ranging from 2.2 to 7.08 Mb and the average GC content was 40.31% with ranging from 35.4 to 47.8%, indicating substantial inter-species or inter-strain variations and similar genomic characteristics. Among these investigated strains, *Bacillus* sp. OxB-1 showed the highest GC content (47.8%), and *B. thuringiensis* and *B. cereus* SJ1 showed lower GC contents (35.4%).

**Table 1:** Genomic features of the chromosome and plasmid of *Bacillus* sp. S3

Features	Chromosome	Plasmid
Genome size (bp)	5,436,472	241,339
Protein-coding genes	5, 131	234
Gene length (bp):	4,638,424	203,412
Gene average length (bp)	904	869
Gene length/genome (%)	85.32	84.28
GC Content in gene region (%)	41.17	37.63
GC content (%)	40.30	36.74
The number of tRNA	104	0
The number of rRNA	36	0
GEIs number	12	3
CRISPR number	4	0
Prophage number	5	0
IS element number	115	48

**Table 2:** Statistical information of the 45 bacterial genomes used in this study

No.	Organism	No. of NCBI accession	Level	Size (Mb)	GC%	No. of Genes	No. of Proteins	rRNA	tRNA
1	*B. asahii* OM18	NZ_CP026095.1	Complete	4.89	37.45	4,824	4,419	54	150
2	*B. bataviensis* LMG 21833	AJLS00000000.1	Contig	5.37	39.6	5238	5,207	–	23
3	*B. cereus* SJ1	ADFM00000000.1	Contig	5.16	35.4	2,192	2,184	–	8
4	*B. cucumis* V32–6	PGVE00000000.1	Contig	5.71	38.6	5,552	5309	10	129
5	*B. dielmoensis* FF4(T)	NZ_CCAD000000000.1	Complete	4.57	40.9	4,433	4184	20	136
6	*B. drentensis* NBRC 102427	BCUX00000000.1	Contig	5.16	38.7	4,998	4851	–	28
7	*B. drentensis* FJAT-10044	LUUU00000000.1	Scaffold	5.3	38.9	5163	4890	51	125
8	*B. firmus* NBRC 15306	BCUY00000000.1	Contig	4.42	41.7	4,515	4170	–	26
9	*B. firmus* NCTC10335	UFTC00000000.1	Contig	4.8	41.7	4,948	4,436	36	108
10	*B. firmus* 14_TX	QNSF00000000.1	Scaffold	5.84	40.8	6009	5,834	23	102
11	*B. glycinifermentans* BGLY	LT603683.1	Complete	4.61	46.1	4,735	4381	24	82
12	*B. glycinifermentans* SRCM103574	CP035232.1	Complete	4.81	46.01	4,948	4,572	25	82
13	*B. licheniformis* ATCC 14580	CP000002.3	Complete	4.22	46.2	4,382	4,219	21	72
14	*B. licheniformis* YNP1-TSU	NZ_CM007615.1	Chromosome	4.25	45.9	4,388	4,227	4	66
15	*B. mesonae* FJAT-13985	LUUQ00000000.1	Scaffold	5.81	40.3	5,553	5,309	40	104
16	*B. mesonae* H20–5	CP022572.1	Complete	5.84	40.4	5,600	5,330	41	109
17	*B. methanolicus* MGA3	CP007739.1	Complete	3.34	38.7	3,355	3,092	27	91
18	*B. niacin* DSM 2923	JRYQ00000000.1	Scaffold	2.2	38.3	2,167	1,922	3	35
19	*B. novalis* NBRC 102450	NZ_BCVP00000000.1	Contig	5.57	39.9	5387	5,212	–	31
20	*B. novalis* FJAT-14227	LUUR00000000.1	Scaffold	5.67	40	5,517	5,247	37	118
21	*B. oceanisediminis* Bhandara28	MBRJ00000000.1	Contig	5.88	40.8	5,925	5,705	33	106
22	*B. soli* NBRC 102451	BCVI00000000.1	Contig	5.46	39.5	5,289	5,109	3	18
23	*B. soli* DSM 15604	NISV00000000.1	Scaffold	5.58	39.7	5,451	5,148	43	92
24	*B. subtilis* subsp. spizizenii str. W23	CP002183.1	Complete	4.03	43.9	4116	3,912	24	77
25	*B. subtilis* subsp. subtilis str. 168	AL009126.3	Complete	4.22	43.5	4,536	4,237	30	86
26	*B. thuringiensis* serovar konkukian str. 97–27	AE017355.1	Complete	5.24	35.4	5,263	5,117	41	105
27	*B. thuringiensis* YBT-1518	CP005935.1	Complete	6	35.4	6371	5,837	45	90
28	*B. vireti* LMG 21834	ALAN00000000.1	Contig	5.28	39.7	5106	5,084	–	21
29	*B. vireti* DSM 15602	LDNB00000000.1	Scaffold	5.31	39.8	5,118	4,794	13	89
30	*B. velezensis* FZB42	CP000560.1	Complete	3.91	46.5	3,892	3,687	29	88
31	*Bacillus* sp. AFS006103	NTXX00000000.	Scaffold	5.18	38.6	5,018	4,840	5	92
32	*Bacillus* sp. OK048	FNHN00000000.1	Scaffold	5.17	38	5,225	5,058	25	115
33	*Bacillus* sp. OV166	FXWM00000000.1	Contig	7.08	38.3	7,182	6,561	60	152
34	*Bacillus* sp. UNC41MFS5	JMLP00000000.1	Scaffold	3.27	38.6	3107	3,014	6	25
35	*Bacillus* sp. UNC438CL73TsuS30	AXVA00000000.1	Scaffold	3.06	39	2,983	2,867	6	41
36	*Bacillus* sp. LF1	CVRB00000000.1	Contig	5.6	38.1	5,524	5277	17	102
37	*Bacillus* sp. FJAT-18017	CP012602.1	Complete	5.27	42.4	5,018	4,825	30	85
38	*Bacillus* sp. FJAT-29814	LMTJ00000000.1	Scaffold	5.89	41.9	5,791	5,596	11	85
39	*Bacillus* sp. X1	CP008855.1	Complete	3.42	38.1	3,433	3,103	36	122
40	*Bacillus* sp. MUM 116	MLYR00000000.1	Contig	5.72	38.4	5,600	5,273	25	165
41	*Bacillus* sp. OxB-1	AP013294.1	Complete	3.59	47.8	3,604	3,438	22	83
42	*Bacillus* sp. WN066	SMYO00000000.1	Contig	6.21	38.6	6,131	5,757	36	151
43	*Bacillus* sp. 7884–1	NPDD00000000.1	Contig	6	38	5,848	5,597	–	46
44	*Bacillus* sp. MRMR6	MSLS00000000.1	Contig	5.44	38.8	5,276	4,978	50	108
45	*Bacillus* sp. S3	CP039727.1	Complete	5.58	40.3	5,131	5344	104	36

“-”: unpublished

Based on BLASTP searches (e value <1e^− 5^), there were 3833 and 2563 CDSs involved in COG database (Figure S2; Table S1) and GO database (Figure S3; Table S2), respectively. A high proportion of genes in COG database were assigned to the general function prediction only (R, 10.3%), amino acid transport and metabolism (E, 8.05%), carbohydrate transport and metabolism (G, 6.15%), energy production and conversion (C, 5.33%), transcription (K, 5.06%), and replication, recombination, and repair (L, 4.6%) categories. In addition, the proteins were distributed to GO database in three functional classification, including “molecular function” (2715), “cellular component” (1001) and “biological process” (3615). Compared to other bacteria, enrichment profiles of *Bacillus* sp. S3 genes assigned to COG functional categories showed an overabundance of genes involved in amino transport and metabolism, carbohydrate transport and metabolism, energy production and conversion. These resistance genes, such as ABC antiporters and Cd^2+^/Zn^2+^/Co^2+^ efflux components (CzcABC, CzcD), were probably important for *Bacillus* sp. S3 in the adaption of specific niche.

In addition, the KEGG pathway database was used to map their corresponding terms on the *Bacillus* sp. S3 genome. A total of 1195 CDSs were assigned to 180 KEGG pathways (Figure S4; Table S3), which could enrich in “Metabolism” (637), “Biosynthesis of secondary metabolites” (285), “Microbial metabolism in diverse environments” (215), “Carbon metabolism” (135), “Biosynthesis of amino acids” (134) and “ABC transporters” (122). The arsenite oxidase (AioB) was assigned to metabolic pathway (ko01100), the phosphate-binding protein (PstS). sn-glycerol-3-phosphate-binding periplasmic protein (UgpB) and phosphate import ATP-binding protein (PstB) were assigned to ABC transporters pathway (ko02010). The copper-ion-binding protein CopZ was assigned to mineral absorption pathways (ko04978). These pathways might play an important role in coping with the metal (loid) toxicity in *Bacillus* sp. S3.

As shown in Figure S5, we identified 237 carbohydrate-active enzymes (CAZymes) in the genome of *Bacillus* sp. S3. Predicted CAZymes were classified into 6 classes, encompassing auxiliary activities (AAs, 4), carbohydrate-binding modules (CBM, 63) carbohydrate esterases (CEs, 39), glycoside hydrolases (GHs, 90), glycosyltransferases (GTs, 39), polysaccharide lyases (PLs, 2). To induce inhibition, the heavy metal (loid) ions may non-specifically bind to regions of CAZymes [24]. This interactions between heavy metal (loid) s and CAZymes need a great deal of energy (e.g. for pumping out intracellular metal ions by ATPases, or for the strengthened expression of metal (loid) resistance proteins, etc.) through increased conversion of carbohydrates [25]. It implied that these enzymes might play an important role in coping with the metal (loid) ions [24].

Notably, large numbers of heavy metal (loid) resistance genes were found to locate on the chromosome rather than plasmid in *Bacillus* sp. S3 (Table 3), which could be used for further analysis of genetic diversity and evolution. Unlike chromosome, the plasmid was found to contain large numbers of hypothetical proteins. In our study, the *aioB* gene encoding Sb(III)/As (III) oxidase was only detected in the chromosome of *Bacillus* sp. S3 and not the other *Bacillus* genomes, giving *Bacillus* sp. S3 the capacity to oxidize Sb(III). Moreover, three *arsB* genes encoding Sb(III)/As (III) efflux pump membrane proteins and *arsC* gene encoding As(V) reductase were located on the chromosome in *Bacillus* sp. S3. As resistance genes (*arsB* and *arsC*) were detected in all comparative *Bacillus* strains, implying that cytoplasmic Sb(III) extrusion was the main As/Sb resistance strategy in *Bacillus* genus. The phosphate (Pi) related genes such as *phoB*, *pstS*, and *phoR* were also identified in *Bacillus* sp. S3, which have been proved to be co-regulated with As (III) oxidation and could be induced by Sb(III) in previous report [26].

**Table 3:** Genes associated with putative heavy metal (loid) s resistance in *Bacillus* sp. S3

Category	Gene ID	Gene	Protein	Function
Arsenate/arsenite detoxification	FAY30_05805	*aioB*	Small subunit of arsenite oxidase	As (III) oxidation
	FAY30_02870	*arsB_123*	Putative arsenical pump membrane protein	As (III) efflux pump
	FAY30_09810			
	FAY30_12790			
	FAY30_09815	*arsC*	Arsenate reductase	As(V) reduction
Copper detoxification	FAY30_07975 FAY30_15235 FAY30_16820 FAY30_16835	*copA_1234*	Copper-exporting P-type ATPase; Lead, cadmium, zinc and mercury transporting ATPase	Cation translocation P-type ATPase
	FAY30_11165 FAY30_16840	*copZ_12*	Copper chaperone	
	FAY30_05265	*cutC*	Copper homeostasis protein	
Chromate detoxification	FAY30_22440	*chrR*	Chromate reductase	
	FAY30_11465 FAY30_20185 FAY30_20190	*chrA_123*	Chromate transport protein	
Cadmium, zinc, cobalt, mercury detoxification	FAY30_09760	*cadC*	Cadmium resistance transcriptional regulatory protein	
	FAY30_09765	*cadA*	Putative cadmium-transporting ATPase	
	FAY30_21075	*zupT*	Zinc transporter	
	FAY30_06055	*zosA*	Zinc-transporting ATPase	
	FAY30_11960	*yeiR*	Zinc-binding GTPase	
	FAY30_17115	*zur*	Zinc uptake regulation protein	
	FAY30_15605	*znuA*	Zinc ABC transporter, substrate-binding protein	
	FAY30_20065	*znuB*	Zinc ABC transporter, permease protein	
	FAY30_08125	*znuC*	Zinc ABC transporter, ATP-binding protein	
	FAY30_20440	*czcD*	Cadmium, cobalt and zinc/H(+)-K(+) antiporter	Cation efflux system protein
	FAY30_10430	*corC*	Magnesium and cobalt efflux protein	
	FAY30_24820	*corA*	Cobalt/magnesium transport protein	
		*merR*	Mercuric resistance operon regulatory protein	
Nickel, molybdenum, detoxification	FAY30_20460	*nikMN*	Nickel transport protein	
	FAY30_20940	*modA*	Molybdate-binding periplasmic protein	
	FAY30_18400	*modB*	Molybdenum transport system permease protein	

### Phylogenetic analysis and ANI calculations

**Fig. 3:**
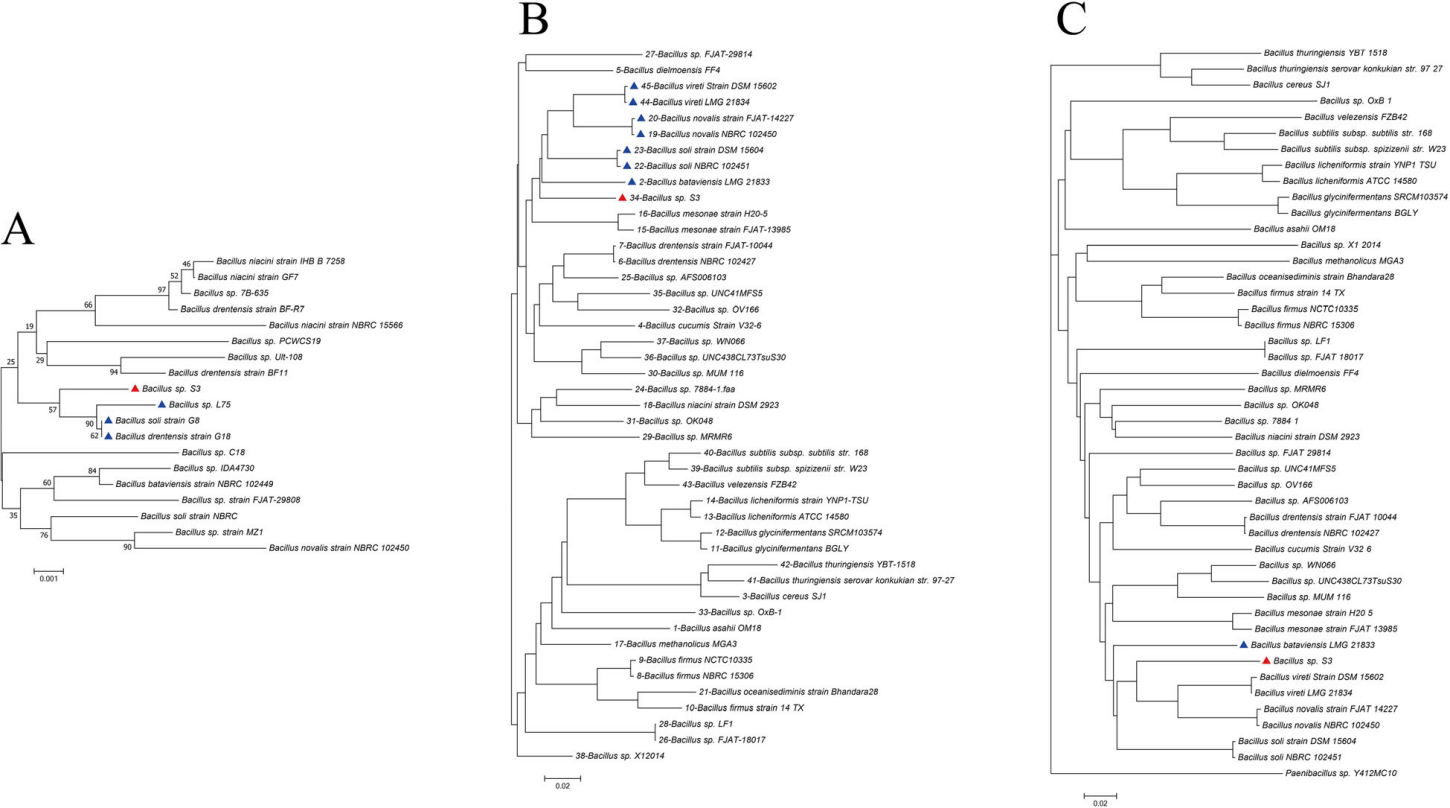
Phylogenetic relationships of 45 *Bacillus* strains. Phylogenetic trees based on (**a**)16S rRNA genes derived from *Bacillus* sp. S3 and other closely-related strains. **b** 554 core genes. Bootstrap values are indicated at each node based on a total of 1000 bootstrap replicates. **c** Whole-genome-based phylogeny trees using a composition vector (CV) approach. *Bacillus* sp. S3 was marked in red blot and other strains that formed a group with *Bacillus* sp. S3 were marked in blue. *Paenibacillus* sp. Y412MC10 was used as an outgroup

To evaluate the phylogenetic relationship, we downloaded 44 *Bacillus* genome sequences (including 16 complete genomes) and their annotations from the NCBI database (Table 2). The phylogenetic tree based on 16S rRNA gene sequences revealed that *Bacillus* sp. S3 belonged to *Bacillus* genus and grouped with *Bacillus* sp. L75, *Bacillus soli* strain G8 and *Bacillus drentensis* G18 (Fig. 3a). The phylogenetic trees based on 554 core genes and whole-genome composition vectors (CVs) were also constructed (Fig. 3b, c). In the two phylogenetic trees, *Bacillus* sp. S3 grouped with *Bacillus bataviensis*, *Bacillus soli*, *Bacillus novalis*, *Bacillus vireti*, indicating that *Bacillus* sp. S3 was closest to the *Bacillus bataviensis*. However, the topologies of the two phylogenetic trees exhibited some differences, suggesting that the flexible genes could be crucial in altering the genome content and shaping the topology of the trees. As shown in Table 4, the closest ANI values 81.51% between *Bacillus* sp. S3 and other reference strains considerably lower than threshold value of 95–96% of the boundary for species circumscription [27]. Data illustrated in Table S4 showed that the dDDH% values of *Bacillus* sp. S3 against all reference genomes ranged from 12.7 to 34%. Therefore, the combination of ANI values and dDDH values showed that *Bacillus* sp. S3 belonged to novel species within the genera of *Bacillus*.

**Table 4:** Average nucleotide identities (ANI) analysis of *Bacillus* sp. S3 and other *Bacillus* species

	S3	LMG 21833	LMG 21834	DSM 15602	NBRC 102451	DSM 15604	NBRC 102450	FJAT-14227	ATCC 14580	YNP1-TSU	OM18	LF1	W23	168	OK048
***Bacillus*sp. S3**	*	81.68	78.48	78.47	78.34	78.37	78.25	78.3	67.49	66.8	69.62	74.47	67.72	67.8	74.02
***B. bataviensis*LMG 21833**	81.34	*	78.7	78.68	78.84	78.84	78.99	78.98	66.61	66.49	69.28	74.06	66.92	66.99	73.69
***B. vireti*LMG 21834**	78.25	78.78	*	**99.99**	79.6	79.61	89.59	89.59	66.72	66.61	68.86	74.48	66.77	66.86	73.94
***B. vireti*DSM 15602**	78.15	78.7	**99.98**	*	79.7	79.7	89.6	89.61	66.75	66.65	68.79	74.42	66.85	66.91	73.99
***B. soli*NBRC 102451**	78.06	78.73	79.74	79.74	*	**99.99**	80.58	80.6	66.51	66.45	69.01	74.81	66.75	66.79	74.28
***B. soli*DSM 15604**	78.39	79.05	79.94	79.92	**99.99**	*	80.76	80.78	67.61	66.9	69.99	75.11	67.9	67.92	74.83
***B. novalis*NBRC 102450**	78	78.97	89.46	89.46	80.6	80.61	*	**100**	66.7	66.6	68.54	74.53	66.69	66.71	74.08
***B. novalis*FJAT-14227**	78.42	79.31	89.58	89.56	80.66	80.7	**99.99**	*	67.63	67.04	69.49	74.92	67.55	67.76	74.43
***B. licheniformis*ATCC 14580**	67.35	67.09	67.24	67.29	67.08	67.16	67.32	67.33	*	**99.57**	66.99	67.12	72.15	72.16	67.13
***B. licheniformis*YNP1-TSU**	66.76	66.55	66.71	66.73	66.54	66.58	66.86	66.88	**99.41**	*	66.6	66.58	71.83	71.75	66.74
***B. asahii*OM18**	70.12	70.21	69.86	69.8	70	70.05	69.79	69.85	67.96	67.09	*	70.07	68.52	68.46	69.93
***Bacillus*sp. LF1**	74.3	74.32	74.64	74.66	75.01	75.04	74.75	74.8	66.83	66.64	69.47	*	67.14	67.19	73.34
***B. subtilis*subsp. str. W23**	67.61	67.52	67.49	67.44	67.38	67.43	67.48	67.54	72.12	71.88	67.83	67.47	*	92.5	67.29
***B. subtilis*subsp. str. 168**	67.67	67.62	67.53	67.47	67.46	67.45	67.52	67.53	72.14	71.82	67.87	67.53	92.23	*	67.57
***Bacillus*sp. OK048**	74.04	73.97	74.26	74.3	74.56	74.59	74.21	74.26	66.58	66.53	69.16	73.35	66.86	66.85	*

Numbers in green denote strains belonging to the same species. The asterisk indicates that the strain is compared to itself, which does not provide valuable information

### Core and pan genomes analyses

**Fig. 4:**
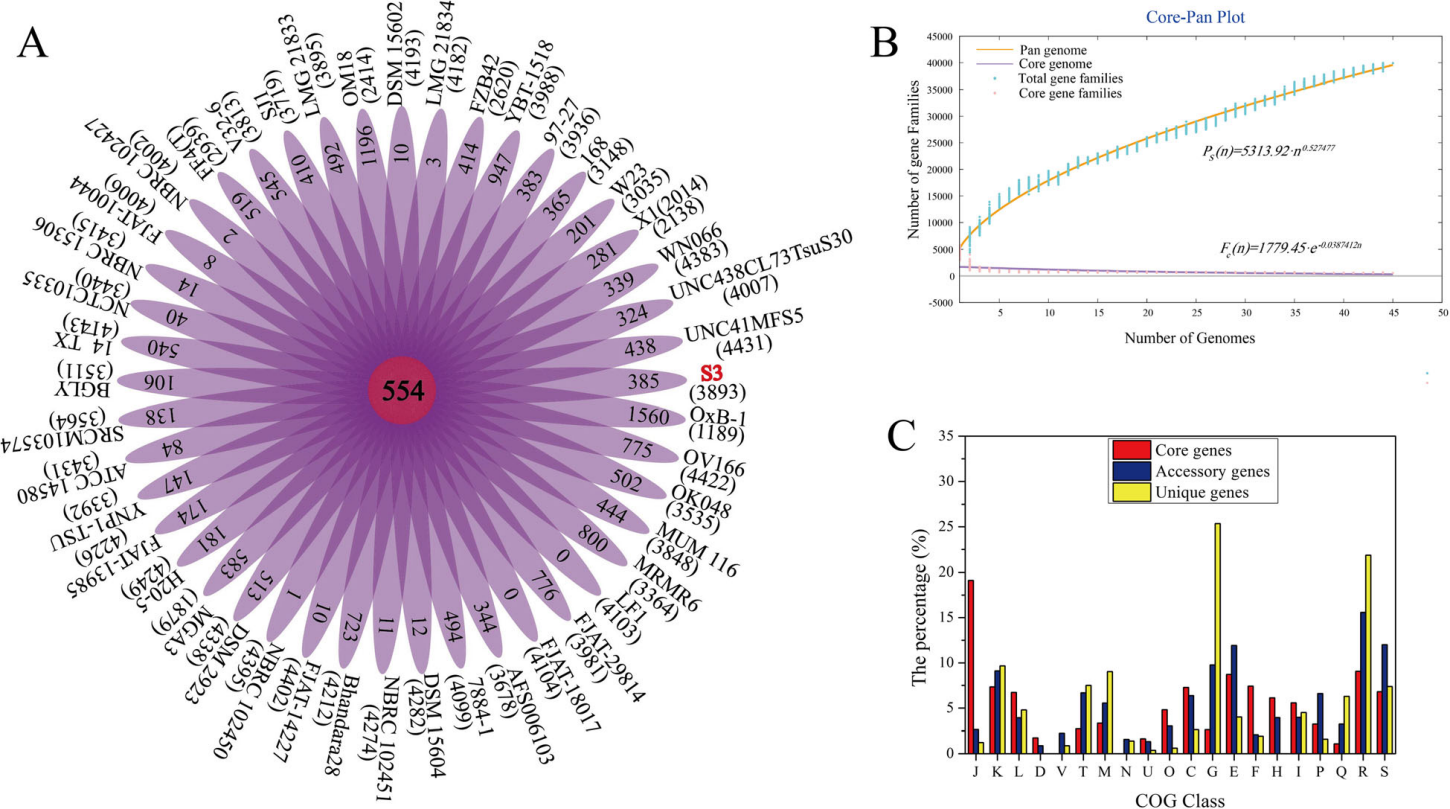
Pan genome analysis of strains within the *Bacillus* genus. **a** Venn diagram displaying numerous core genes and flexible genes for each of the 45 *Bacillus* strains. Each purple oval represented a strain. The numbers of orthologous coding sequences (core genes) shared by all strains were represented in the center. Numbers in nonoverlapping portions of each oval showed the numbers of unique genes to each strain. The total numbers of accessory genes within each genome were listed below the strain names. *Bacillus* sp. S3 was marked in red blot. **b** Mathematical modeling of the pan-genome and core genome of *Bacillus* strain. **c** Proportion of genes enriched in the clusters of orthologous groups (COG) categories in unique genes, accessory genome, and pan-genome according to COG database

To clarify the genomic features specific to each *Bacillus* strain, all genes from tested *Bacillus* strains were described by MP method in the pan-genome analysis pipeline with a 50% cut-off for protein sequence identity. There were total 39,933 orthologs protein coding sequences in the pan genome for the *Bacillu*s genus (Fig. 4a). Of these orthologs genes, 554 (1.38% of total pan genome) were identified as core conserved genes, and 16,234 were identified as strain-specific genes. The numbers of accessory genes varied from 1189 to 4431 (mean 3693) and the *Bacillus* sp. S3 had 3893 accessory genes. Accessory gene is known as indispensable orthologs, whose variability indicates the flexibility of genome structure [28]. After comparing strain-specific genes, the variability in the total number of strain-specific genes ranged from 0 to 1560 genes (mean 360). *Bacillus* sp. OxB-1 had the highest amount of these (*n* = 1560), reflecting the greatest difference with other tested genomes.

Previous reports stated that a mathematical extrapolation of the pangenome was positively reliable when sufficient genomes (> 5) were involved [29]. The deduced power law regression function [*P*_*s*_(*n*) = 5313.92*n*^0.527477^] revealed that the pan-genome of *Bacillus* had a parameter (γ) of 0.527477 (0 < γ < 1), implying a stabilized core structure and an open pan-genome of *Bacillus* strains (Fig. 4b). Thus, the new orthologs were intuitively observed after addition of more genomes to the group. Furthermore, the extrapolated curve of the core genome presented a steep slope according to the exponential regression [*F*_*c*_(*n*) = 1779.45e^-0.0387412*n*^] (Fig. 4b). The addition of an extra genome would not significantly alter the size of the core genome due to the numbers of core genes were relatively stable [25].

These core genes shared with all *Bacillus* genomes also could be classified into different COG categories (Fig. 4c), which was agreed with previous reports that larger prokaryotic genomes tended to pile up genes directly or indirectly involved in different metabolisms [30]. The KEGG annotation of 385 specific genes of *Bacillus* sp. S3 showed that 10 genes involved in the environmental information processing, including one cobalt transport system protein-encoding gene *corA* and one As (III) efflux pump membrane proteins-encoding gene *arsB*. Further, small numbers of strain-specific genes (< 40%) were assigned to the COG categories for the *Bacillus* sp. S3, which were mainly found to be enriched in phosphotransferase ABC-type metal ion transport systems. The result revealed specific adaptive strategies of *Bacillus* sp. S3 in response to harsh eco-environments.

### Mobile gene elements in *Bacillus* genomes

The presence of the majority of genomic islands (GEIs) in *Bacillus* sp. S3 and other comparative strains rendered clue about the genomic plasticity of these isolates (Table S5). We identified 20/5/15 GEIs in *Bacillus* sp. S3 through three methods. These GEIs were possibly conducive to the integrated pool of transposase. Analysis of transposable elements showed that numerous insertion sequences (IS) were distributed over the genomes and plasmids of *Bacillus* strains, harboring IS1, IS2, IS3, IS4, IS5, IS21 and IS256. The majority of IS could magnify the size of genome, and result in frequently genomic exchange with other community members [31]. As shown in Table S5, all *Bacillus* genomes could be served as feasible targets of phage infections. A total of 5 intact (100% score) prophage regions were predicted in the genome of *Bacillus* sp. S3, their information containing size: 11,485 bp, 5841 bp, 8630 bp, 8468 bp, 7959 bp; coding sequence: 11, 7, 6, 10, 8; GC content: 42.84, 38.02, 35.25, 36.25, 33.84% (Figure S6). Furthermore, the number of CRISPRs varied from 0 to 15 per strain and CRISPR locus absolutely scattered the chromosome. Four confirmed CRISPR locus were detected in *Bacillus* sp. S3 with 39, 20, 60, 4 spacers.

### Comparative genomic analyses of *Bacillus* genus

**Fig. 5:**
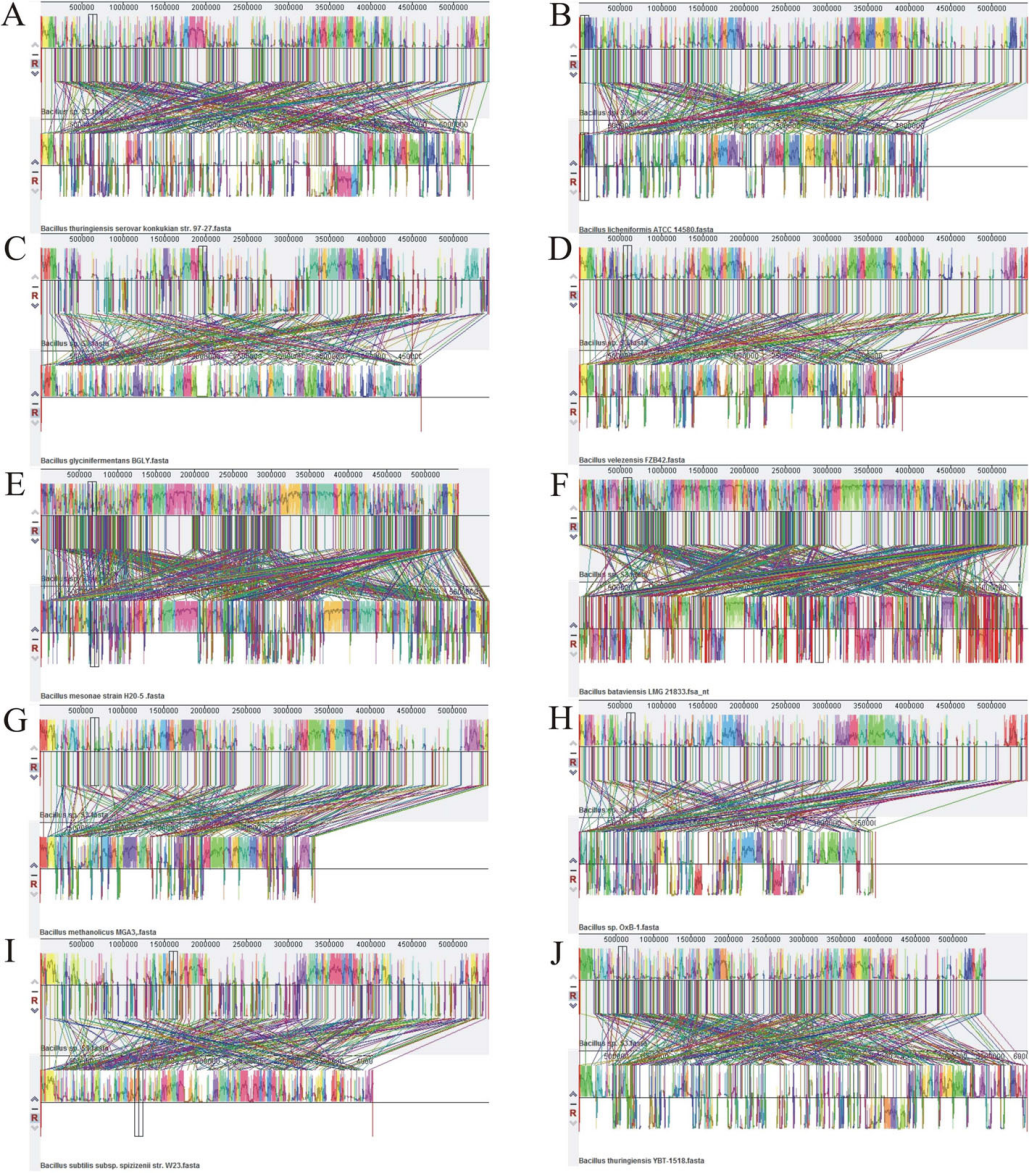
Mauve alignment of *Bacillus* sp. S3 with its closer *Bacillus* genomes. *Bacillus thuringiensis* serovar konkukian str. 97–27 (**a**); *Bacillus licheniformis* ATCC 14580 (**b**); *Bacillus glycinifermentans* BGLY (**c**); *Bacillus velezensis* FZB42 (**d**); *Bacillus mesonae* H20–5 (**e**); *Bacillus bataviensis* LMG 21833 (**f**); *Bacillus methanolicus* MGA3 (**g**); *Bacillus* sp. OxB-1 (**h**); *Bacillus subtilis* subsp. spizizenii str. W23 (**i**); *Bacillus thuringiensis* YBT-1518 (**j**). Boxes of different colors demonstrate the sequence coordinates and the conserved segments represented LCBs (or locally conserved regions). The LCBs above and below the reference line of the consistent color represent the orientation of the LCBs relative to the reference sequence for each genome. White areas represent possibly contain genome-specific sequence elements and those genomic positions that did not adequately align between the selected genomes

Mauve has been used for constructing multiple genome alignments in large-scale evolutionary events such as genome rearrangement, inversion, and other recombination [32]. In order to prove the extent of genomic shuffling, the whole genome sequence of *Bacillus* sp. S3 was individually compared with the other 10 complete genomes using mauve with default settings. As shown in Fig. 5, synteny maps of the 10 complete genomes were inspected, and represented large-scale blocks of inversions and several crisscrossing locally collinear blocks (LCBs). 615 LCBs with a minimum weight of 45 were exhibited between *Bacillus* sp. S3 and *B. bataviensis* LMG21833, and other comparative information was also observed. Mauve analyses showed *Bacillus* sp. S3 had the highest synteny with *B. bataviensis* LMG21833 genome. Compared to *B. bataviensis* LMG21833, other strains exhibited more rearrangements, insertions and deletions. Conserved structural synteny and lack of inversions and rearrangements were observed among members of *Bacillus* group, suggesting that the large-scale evolutionary events were occurred at the genus level [24]. This was in agreement with genome distance in phylogenetic tree.

### The origin and evolution of Sb(III)-related genes

To explore the origin and evolution of Sb(III)-related genes in *Bacillus* sp. S3, typical genes including *aioB*, *arsB* and *arsC* were selected for analysis. The deviant G + C contents between gene and the genome could be used as a detection method of HGT [27]. Herein, we detected the G + C contents of Sb(III)-related genes and their corresponding genomes in *Bacillus* strains. As shown in Figure S7, the average GC contents of the Sb(III)-related genes were different from that of their corresponding genomes in *Bacillus* strains. It is worth noting that the *aioB* gene was a specific gene of *Bacillus* sp. S3, whose GC content was significantly higher than that of the genome (44.64 vs. 40.3).

The overall origin and evolution of Sb(III)-related genes in *Bacillus* genus were inferred by phylogenetic trees with NJ, ML and UPGMA methods. We speculated that the *aioB* gene in *Bacillus* sp. S3 derived from the evolution of 2Fe-2S ferredoxin (Figures S8, S9 and S10). To further elucidate the evolution of *arsB*_123 and *arsC*, phylogenetic trees based on ArsB and ArsC proteins were constructed (Figures S11, S12, S13, S14, S15 and S16). As shown in Figure S11, S12, S13, the *arsB_123* genes of *Bacillus* sp. S3 formed separate 3 groups and a monophyletic clade, indicating that *Bacillus* sp. S3 might obtain *arsB_123* genes from *B. bataviensis* LMG21833, *B. vireti* and *B. drentensis*. Notably, the *arsB* gene of *Bacillus cereus* grouped with *Klebsiella pneumoniae* (Figure S11 and S12), the *arsB* gene of *Bacillus megaterium* grouped with *Paenisporosarcine* sp. HGH0030 (Figure S11 and S13). The results revealed that *Bacillus* species could acquire *arsB* gene from other genera via HGT. As shown in Figures S14, S15 and S16, the phylogenetic analysis revealed that the *arsC* gene of *Bacillus* sp. S3 only grouped with *B. bataviensis*, implying that *B. bataviensis* was likely donors of the *arsC* gene. Meanwhile, the *arsC* gene of *Bacillus* sp. 7884–1 grouped with *Rhodococcus qingshengii*, *Bacillus oceanisediminis* grouped with *Xanthomonas citri* (Figure S15), indicating that Sb(III)-related genes of *Bacillus* species might be acquired via HGT events from other genera. The results suggested that the *arsB* gene and *arsC* gene of *Bacillus* sp. S3 might originate from a common ancestor with *B. bataviensis*.

### Assessment of functionality of heavy metal (loid)s-related genes

**Fig. 6:**
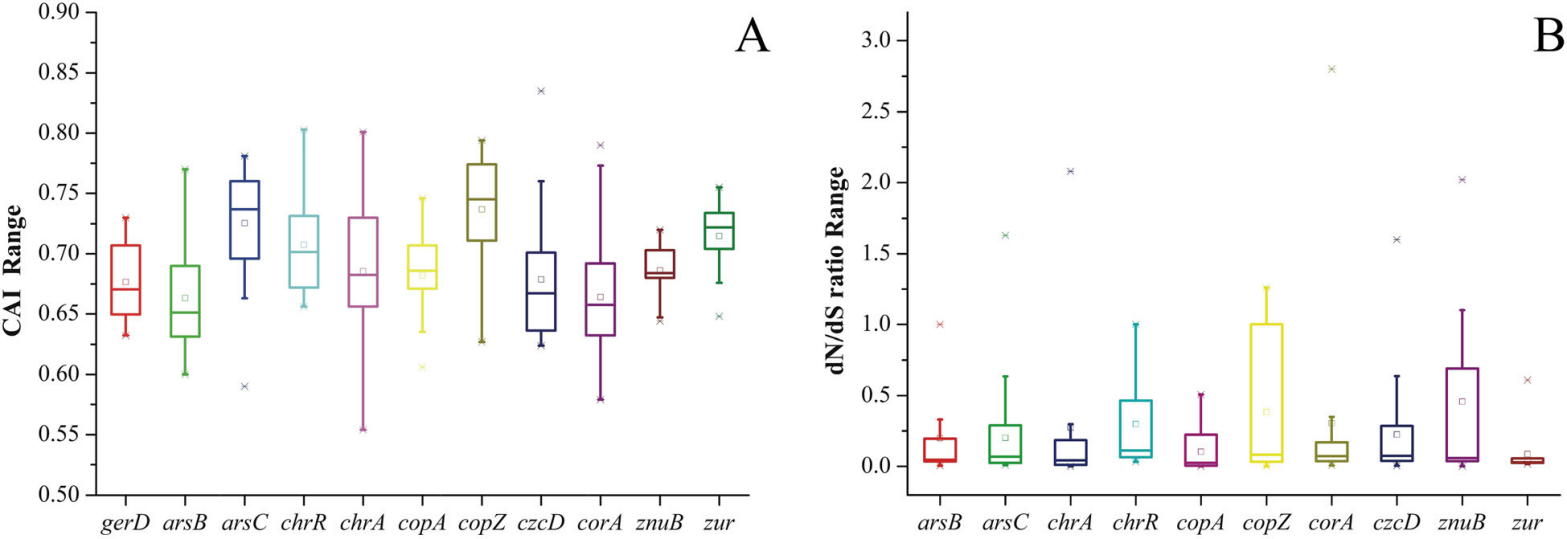
Ranges of CAI values of different metal resistance genes of *Bacillus* species with *gerD* gene as a reference (**a**). Distributions and ranges of selection pressure on various metal resistance genes within the *Bacillus* genus (**b**)

Previous reports showed that the CAI (codon adaptation index) was a numerical estimator of gene expression level, due to highly expressed genes in bacteria were prone to magnify stronger codon bias [33]. The CAI value varies from 0 to 1.0, and higher CAI value indicates a higher expression level [34]. In order to indirectly assess the functionality of metal (loid) resistance genes, the appraisal of the strength of natural selection was performed, along with the CAI values of these genes. Putative highly expressed (PHX) genes associated to metal (loid) resistance in the *Bacillus* genus were inferred, where *gerd* gene encoding spore germination protein was used as a reference. As shown in Fig. 6a, the CAI values of these above-mentioned metal (loid) s resistance genes were calculated. The cutoff values were indicated with average CAI values of *gerd* genes in each species. Our result showed that only about 8% of the metal (loid) resistance genes were predicted to be PHX genes that greater than 0.75, while CAI values of lots of genes ranged from 0.4 to 0.8. The PHX genes lead to stronger codon bias than those of low expressed level genes due to codon translational selection.

A gene in the node or tip of a given tree was considered under diversifying selection (*dN*/*dS* > 1), evolving neutrally (*dN*/*dS* ≈ 1) or purifying selection (*dN*/*dS* < 1) using the likelihood ratio test after adjusting for multiple testing (*P* value < 0.1) [33]. As shown in Fig. 6b, 96.3% of 10 selected genes associated metal (loid) had a ratio of nonsynonymous substitutions (*dN/dS* < 1), implying that these genes were indispensable factor for the above-mentioned *Bacillus* species under the pressure of purifying selection. Only an *arsC* gene from *B. bataviensis* LMG21833 (*dN/dS* = 1.63), double *chrA* genes from *B. niacin* DSM 2923 and *B. liceniformis* ATCC 14580 (*dN/dS* = 2.08, *dN/dS* = 1.28), a *copZ* gene from *B. firmus* NCTC 10335 (*dN/dS* = 1.28), a *corA* gene from *Bacillus* sp. LF1 (*dN/dS* = 2.80), double *znuB* genes from *B. soli* 15604 and *B. bataviensis* LMG21833 (*dN/dS* = 2.02, *dN/dS* = 1.1) showed *dN/dS* > 1, suggesting that they might be under diversifying selection. Furthermore, the lowest *dN/dS* ratio was remarked for *copA* gene (average *dN/dS* = 0.08) and the *zur* gene (average *dN/dS* = 0.09), demonstrating strong purifying selection. Additionally, these genes including *arsB* from *Bacillus mesonae* H20–5, *chrR* from *Bacillus mesonae* H20–5 and *Bacillus* sp. S3, *copZ* from *B. thuringiensis* 97–27, *Bacillus glycinifermentans* BGLY and *B. oceanisediminis* Bhandara28, *corA* from *B. firmus* 14_TX, showed *dN/dS* ratio = 1.0, indicating that selection force had little effect on them. The results showed that metal (loid) resistance genes had universally low *dN/dS* ratios and high CAI values, indicating that their functions played a role in supporting the growth of *Bacillus* species in response to harsh environments [34].

### Transcriptional expression analysis in *Bacillus* sp. S3 with or without Sb(III)

**Fig. 7:**
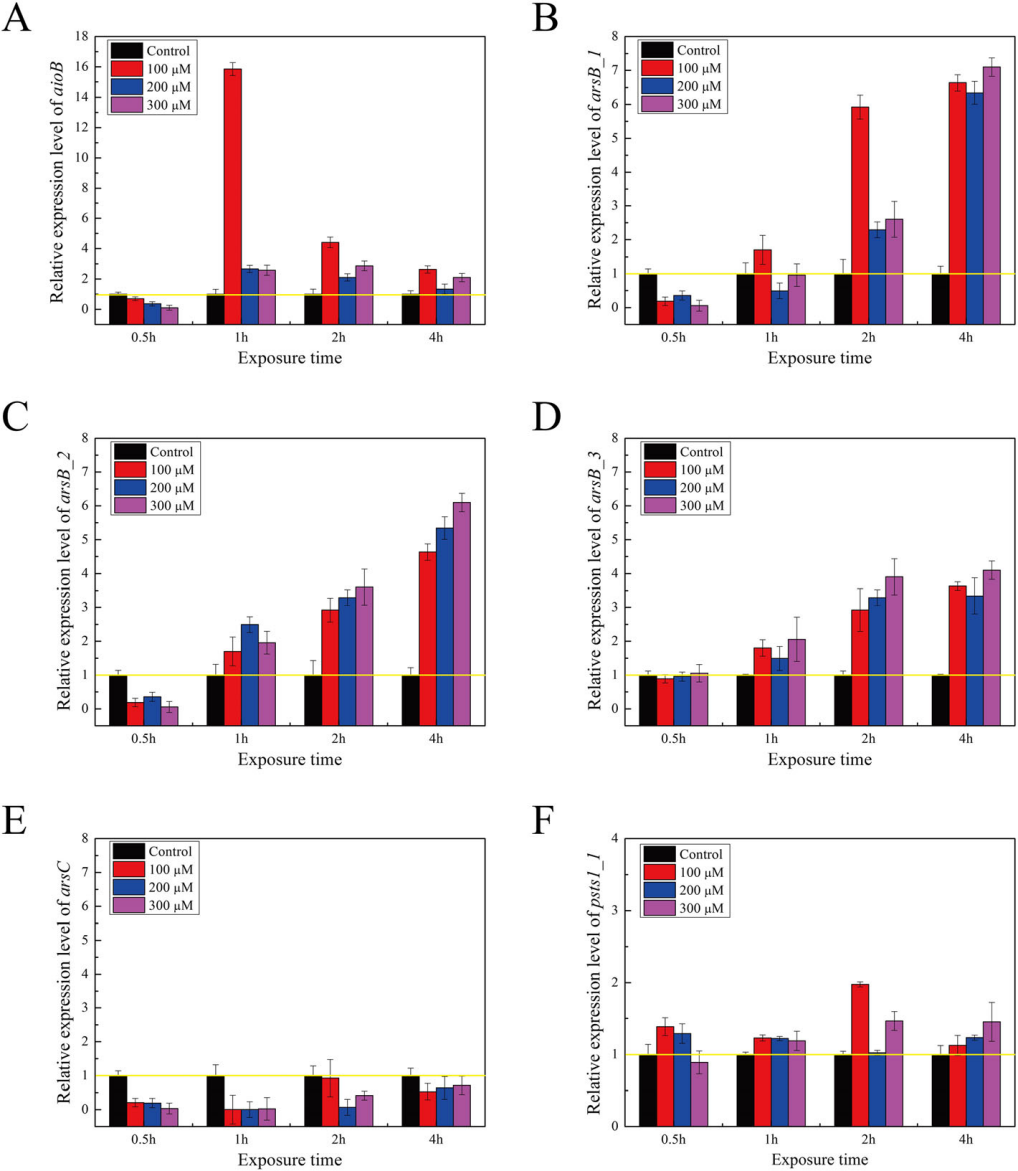
Real-time quantitative PCR analysis of the genes encoding proteins involved in antimonite/arsenate oxidation (**a**), antimonite/arsenite resistance (**b**, **c**, **d** and **e**) and phosphate metabolism (**f**). Data shown as the mean of three replicates with the error bars representing ± SD

To gain the insights into the role of Sb(III)-related genes, the expression levels of *aioB*, *arsB_123*, *arsC* and *psts_1* were investigated by RT-qPCR. Primers used in the study were listed in Table S6, where 16S rRNA gene was used as an internal reference. As shown in Fig. 7, the transcriptional expression levels of most of genes were up-regulated by Sb(III) except for *arsC*. Although the expression levels of *aioB* and *arsB_123* in exposure of 0.5 h Sb(III) were down-regulated, the expression levels were up-regulated after 1 h, 2 h and 4 h, respectively. Compared to uninduced culture, the expression level of *aioB* gene increased 15.8, 4.4 and 2.6 folds with 100 μM Sb(III) from 1 to 4 h. Nevertheless, when the *Bacillus* sp. S3 was exposed to high Sb(III) concentration (200 and 300 μM), the expression level of *aioB* gene was up-regulated 2.6, 2.1, 1.3 and 2.5, 2.8, 2.1 folds from 1 to 4 h, respectively. Thus, the expression level of *aioB* was independent of Sb(III) concentration and stress time. As shown in Fig. 7, the expression levels of *arsB_123* (2 h and 4 h) were remarkably up-regulated. The expression levels of *arsB_123* and *psts_1* were up-regulated along with the increase of Sb(III) concentration. The increased expression levels of *aioB* and *arsB_123* in presence of Sb(III) suggested that Sb(III) could stimulate the expression levels of As (III)/Sb(III) resistance genes, which might act synergistically to release the toxicity of Sb(III) in *Bacillus* sp. S3.

## Discussion

In the previous study, *Bacillus* sp. S3 showed high Sb(III) oxidation activity and multiple heavy metal (loid) s resistance capability [18]. The morphological characterization of *Bacillus* sp. S3 in presence of heavy metal (loid) s was performed in this work. The SEM results showed that the elevated levels of heavy metal (loid) ions might suppress the secretion of extracellular polymers substance and normal metabolism (Fig. 1). The smallness of bacterial cells provided a large contact interface, which would facilitate the interaction between heavy metal (loid) s and biosorption process of *Bacillus* sp. S3. The EDS spectra results revealed that *Bacillus* sp. S3 might further absorb Pb (II) compared with other heavy metal (loid) s, such as extracellular adsorption and surface complexation [9].

Sb-oxidizing bacteria can convert Sb(III) to the less toxic Sb(V), which is very important for environmental Sb bioremediation [14]. In this study, the newly sequenced *Bacillus* sp. S3 represented a complete genome, including a circular chromosome and a circular plasmid. To understand the molecular mechanisms that Sb(III) oxidation and resistance, a series of Sb(III)-related genes in *Bacillus* sp. S3 genome were mined, such as *aioB*, *arsB*, *arsC* and Pi-related genes. The *aioAB* genes encoding As (III) oxidase were responsible for Sb(III)/As (III) oxidation, which could convert the more toxic Sb(III) to the less toxic Sb(V) in the periplasm [12]. The data we presence demonstrated a first glimpse into the *aioB* gene encoding arsenate oxidase in *Bacillus* genus. These Sb(III)-related genes of *Bacillus* sp. S3 might play a key role in coping with Sb(III)-polluted sites. Recently, the cytoplasmic Sb(III) oxidase AnoA was identified and characterized in *A. tumefaciens* GW4 by comparative proteomics and RT-qPCR [22]. Compared to *A. tumefaciens* GW4, *Bacillus* sp. S3 has remarkably higher Sb(III) resistance and Sb(III) oxidation ability. However, the *anoA* gene was not found in *Bacillus* sp. S3, indicating other unknown mechanisms. Of course, the genome of *Bacillus* sp. S3 was also harbored a high numbers of other heavy metal (loid) s resistance genes, since many contaminated sites contain multiple heavy metal (loid) s [16]. These results revealed various adaptative mechanisms of *Bacillus* sp. S3 to survive in metal-contaminated environments. It has been reported that resistance genes in response to nitrate and heavy metals were propelled by harsh eco-environments, which involved in defense and repair mechanisms for dealing with heavy metals [10].

Although 16S rRNA gene has been conventionally used for assessing bacterial taxonomy and phylogeny, there were still controversy and uncertainty based on only one gene [35]. To implement the taxonomic classification of *Bacillus* sp. S3 in *Bacillus* genus, the phylogenetic trees based on 554 core genes and whole-genome were constructed. The phylogenetic trees based on 554 core genes and whole-genome showed that *Bacillus* sp. S3 was closest to the *Bacillus bataviensis*. To confirm the findings from the phylogenetic analysis, the ANI and dDDH% analyses were performed. ANI is the most widely accepted bioinformatics tool that evaluate the genomic distance and delineate species in evolutionary progress, overcoming the difficulty of conventional deviations caused by evolutionary mutation rate and HGT events [36]. Besides, the value of 70% dDDH was the recommended standard for species delineation of bacteria, corresponding tightly to 95% ANI [37]. The results of ANI and dDDH values indicated that *Bacillus* sp. S3 represented a novel species.

It is well recognized that bacteria genomes have notable genome plasticity by several elements of HGT events, known as mobile gene elements (GEIs, IS, Prophages and CRISPRs) and plasmids [31]. The results showed that the majority of MGEs were distributed on chromosomes within the *Bacillus* genus. MGEs were key driving forces of genome evolution and played a pivotal role in HGT events [11], indicating that high genomic plasticity in *Bacillus* genus was extended to potential strategies to cope with high metal (loid) ion concentrations of their natural habitats. GEIs, which have been committed to provide antibiotic resistance to the host bacteria, were generally divided into 4 categories based on their function, including resistance island (RIs), virulence genes, metabolic islands, and symbiotic island (SIs) [38]. These islands promoted symbiotic integration of the host with other microorganisms [39]. CRISPR-Cas system is a type of adaptive immunity in bacteria and archaea, which protect them against invading genetic elements [40]. Our findings suggested that the *Bacillus* genus could trigger various defense mechanisms against the invasion of exogenous DNA for maintaining the stability of their genetic architecture during the evolution.

Genome evolution could be driven by the acquisition and loss of genes, which was conducted by HGT, genomic reshuffling and natural selection [41]. A combination of several approaches was implemented to discover putative HGT, since it was difficult to identify via either phylogenetic analysis or deviant G + C content [5]. Our results suggested that the prosperus branch of the *arsB_123* genes of *Bacillus* sp. S3 near the base of the *B. bataviensis* LMG21833, *B. vireti* and *B. drentensis,* and the *arsC* gene grouped with *B. bataviensis*. The results suggested that the *arsB_123* genes and *arsC* gene in *Bacillus* sp. S3 were acquired via HGT from other *Bacillus* species, including *B. bataviensis*, *B. vireti* and *B. drentensis*. Such results were found to be consistent with a previous study which examined evolution of *ars* genes in *Pantoea* spp., in which bacterial genes related to As resistance and detoxification might be acquired via HGT [42]. A previous study showed that a high numbers of metal resistance genes of *C. testosteroni* S44 shared highest similarities with *C. testosterone* KF-1 or *C. testosterone* CNB-2, but not with other genera [16]. However, the *arsC* gene of *Bacillus* sp. 7884–1 might be acquired via HGT events from *Rhodococcus qingshengii* (Figure S15), and the *arsB* gene of *Bacillus cereus* might be acquired via HGT events from *Klebsiella pneumoniae* (Figure S11). The results suggested that Sb(III)-related genes might be acquired from other genera during evolution of the *Bacillus* genus.

Transcriptional patterns of the different Sb(III)-related genes were quite different, indicating the various response of *Bacillus* sp. S3 to Sb(III) detoxification. In the case of Sb(III)-related genes, *aioB*, *arsB_123* and *psts_1* were up-regulated by Sb(III), while the *arsC* gene was down-regulated by Sb(III). On the contrary, with the addition of Sb(III), the *arsC1* and *arsC2* genes showed 2.9 and 4.7 folds up-regulation in *Agrobacterium tumefaciens*, respectively [22]. It has been reported that *aioA* expression was not induced by Sb(III) in *A. tumefaciens* GW4 [11]. In contrast, our results showed that the expression level of *aioB* was up-regulated from 1 to 4 h compared to uninduced cell, indicating that the *aioB* gene was induced by Sb(III) and played a putative part in oxidizing Sb(III). Nevertheless, 0.5 h Sb(III) exposure showed that the expression level of the *aioB* gene was down-regulated during the earlier time points. It was noteworthy that the expression level of the *aioB* gene in higher Sb(III) concentration (200 and 300 μM) notably lower than 100 μM Sb(III), indicating that the higher Sb(III) concentration could inhabit *aioB* expression. These results were basically consistent with the previous reports that the excessive As (III) treatment could inhibit the *aioAB* expression [12]. In addition, the expression level of *psts_1* gene involved in phosphate metabolism and co-regulated the *aioAB* genes was induced by Sb(III), suggesting that *Bacillus* sp. S3 required the DNA repair and amino acids synthesis processes in response to Sb(III) by enhancing production of Pi and phosphoribosyl pyrophosphate [22].

## Conclusion

In this study, we sequenced a hyper Sb(III) oxidation strain *Bacillus* sp. S3 and performed comparative genomic study of the *Bacillus* group, representing substantial improvements over previously published results. The majority of genes encoding metal (loid) resistance proteins and MGEs were discovered in *Bacillus* sp. S3, which could adapt to metal (loid)-contaminated environments. Meanwhile, there was an arsenate oxidase AioB in the *Bacillus* sp. S3, which could play a key role in the process of Sb(III)-oxidizing. Notably, *Bacillus* sp. S3 was identified as a new species by phylogenetic trees and ANI analysis. The origin and evolution analysis of Sb(III)-resistance genes was carried out. In addition, Sb(III)-related genes in the *Bacillus* sp. S3 were induced by Sb(III) using RT-qPCR, indicating these genes occupied a significant position in alleviating the toxicity of Sb(III). These findings could improve our understanding of the genomic characteristics and evolutionary relationships among the *Bacillus* genus. As a consequence of the lack of comprehensive analysis with respect to genetic expression and regulation by *Bacillus* sp. S3, the molecular basis of microorganism-Sb(III) needs to be further elucidated in the near future through gene knockout and protein characterization.

## Methods

### Bacterial strain and culture conditions

*Bacillus* sp. S3 was previously isolated from an antimony-mine area, in Hunan province, China [17]. The bacterial cells of *Bacillus* sp. S3 were aerobically grown in 50 mL Luria broth (LB) medium (10.0 g/L tryptone, 5.0 g/L yeast extract, and 5.0 g/L NaCl, pH 7.0–7.2) with shaking at 150 rpm at 28 ± 2 °C. The bacterial growth was measured using ultraviolet-visible spectrophotometer (Shimadzu UV-2550, Japan) by recording optical density at 600 nm (OD_600_). To determine the hazardous effects of different heavy metal (loid) s on the bacterial growth, different concentrations of C_8_H_4_K_2_O_12_Sb_2_·3H_2_O, NaAsO_2_, CdCl_2_, K_2_CrO_4_, PbNO_3_, Cu (SO)_4_, ZnCl_2_ was added to the culture aliquots [18]. The total concentrations of Sb(III), As (III), Cd (II), Cr (VI), Pb (II), Cu (II) and Zn (II) in each treatment were 100, 1000, 50, 200, 500, 800 and 80 μM, respectively.

### Scanning electron microscopy (SEM) and energy dispersive X-ray spectroscopy (EDS) analysis

Bacterial cells with different metal treatments were firstly harvested in the exponential phase at 4 °C at 4000×*g* for 10 min when the OD_600_ reached 0.8, which were then fixed at 4 °C in 2.5% glutaraldehyde for about 2 h. After being washed twice with phosphate buffer saline (PBS, 0.1 M and pH 7.0) and dehydrated by ethanol (30–100%) for 20 min, the cells samples were dehydrated using a lyophilizer and coated with a thin layer of platinum via vapor deposition. The surface morphology and property of the *Bacillus* sp. S3 cell were analyzed using a SEM (Helios NanoLab G3 UC, Thermo Fisher Scientific, Czech; accelerating voltage: 15 kV) equipped with an energy dispersive X-ray analyzer (X-stream-2; Oxford instruments, Oxford, UK).

### Whole-genome sequencing, assembly and annotation

The genomic DNA of *Bacillus* sp. S3 was extracted using an E.Z.B.A Bacterial DNA Kit (Omega, Bio-Tek, USA) according to the manufacturer’s instructions. The strategy of whole genome sequencing was used a combination of Illumina HiseqXten (Illumina Inc., San Diego, CA, USA) and Pacific Biosciences Sequel sequencing platform (Pacific Biosciences, Menlo Park, CA, USA), and the PE sequence data from the Illumina platform was used to proofread PacBio assembly sequence. Illumina libraries were prepared using NEXTflexTM Rapid DNA-seq Kit (BIOO Scientific Crop., Austin, TX, USA) in terms of the included instructions. A 10-kb SMRT bell library was prepared from sheared genomic DNA using a 10-kb template barcoded library preparation workflow. Single Molecule Real Time (SMRT) sequencing was conducted on a PacBio Sequel sequencing platform using the SMRT v3.0 cell. For the *Bacillus* sp. S3 strain, a total of 88,313 clean reads with an average length of 9904 bp and an N50 size of 13,621 bp were generated (Table S7, S8, and S9).

De novo assembly of the PacBio read sequences was performed using continuous long reads (CLR) following the Hierarchical Genome Assembly Process (HGAP4) workflow (PacBioDevNet; Pacific Biosciences) as available in SMRT Link [43]. HGAP 4 consists of preassembly, de novo assembly with Celera Assembler, and assembly polishing with Quiver. To improve the accuracy of the assembly genome, four rounds of iterative error correction were carried out using the Illumina clean data by in house script. The final assembly generated a circular genome sequence with gapless. The circular maps of chromosome and plasmid were generated using the Circos software (version 0.64) [44]. The genome and plasmid sequences of *Bacillus* sp. S3 were deposited in the NCBI database under the accession numbers CP039727.1 and CP039728.1, respectively.

Protein-coding regions in the assembled sequences were predicted using Prodigal [45]. tRNA and rRNA were separately identified by tRNA-scan and RNAmmer, respectively [45]. Genome annotation was carried out by a command line software tool: rapid prokaryotic genome annotation (Prokka) [46]. The genes functions were determined against the NCBI non-redundant (NR), Gene Ontology (GO), Clusters of Orthologous Groups (COG), and Kyoto Encyclopedia of Genes and Genomes (KEGG) databases with *E*-value cut-off set to 1e^− 5^ and subsequent filtering for the best hit [47].

### Phylogenetic analysis and average nucleotide identity (ANI)

To perform the comparative analysis, 44 most closely related species and representative species were retrieved from the GenBank database by BLASTP search (Table 2). Three different datasets of representative markers, 16S rRNA sequences, core genes and whole genomes were used to construct phylogenetic trees. We obtained the 16S rRNA gene sequences of some strains closely related to *Bacillus* sp. S3 by BLASTP search against the NCBI database. The phylogenetic tree of *Bacillus* sp. S3 based on the 16S rRNA gene sequences was constructed using the NJ method in MEGA v7.0 with 1000 bootstraps replications [48]. Phylogeny based on only one common gene may result in bias; therefore, we constructed the phylogenetic tree based on the core genes, which were shared by the comparative strains (see ‘Comparative genomics analysis’). The core genes of the 44 *Bacillus* strains were fetched by Bacterial Pan Genome Analysis (BPGA) software [49]. The multiple sequences alignments of copy core genes was performed using MUSCLE software [50] and the phylogenetic tree was generated using the NJ method in MEGA v7.0 with 1000 bootstraps replications. In addition, the phylogenetic tree based on whole-genomes of *Bacillus* strains was also constructed in our study, in which *Paenibacillus* sp. Y412MC10 strain was designed as an outgroup [51]. The average nucleotide identity (ANI) values between the newly sequenced *Bacillus* sp. S3 genomes and the representative genomes of *Bacillus* spp. were calculated using the web server JSpecies1.2.1 [32] based on a BLAST algorithm and tetranucleotide frequency correlation coefficient (Tetra) with default parameters [52]. In addition, the DNA-DNA hybridization (DDH) values were calculated using Genome-to-Genome Distance Calculator (GGDC) [53].

### Comparative genomics analysis

BPGA was used to extrapolate pipeline pan-genome models with default parameters, and all of orthologous groups among testing *Bacillus* genus were identified [49]. The core genome is the common set of shared genes among all testing strains, the pan genome is the entire set of genes within test genomes, the accessory genome is the set of genes shared with more than two but not all testing strains and unique genes is the set of genes in each strain not shared with other strains [47]. The details of the strains used were listed in Table 2. Furthermore, synteny maps were generated to unravel the degree of rearrangements (insertions, deletions, duplications) by identifying conserved LCBs among genomes. Multiple alignments of *Bacillus* sp. S3 and 10 selected *Bacillus* genomes were also performed using the Mauve Genome Alignment v2.3.1 with the progressive Mauve algorithm [54].

### Genes for heavy metal (loid) s resistance

The genes related to the resistance of Sb(III) and other heavy metal (loid) s in the *Bacillus* sp. S3 genome and other comparative *Bacillus* genomes were identified by performing BLASTP searches against the BacMet database [55]. Subsequently, each Sb(III) resistance gene was compared on NCBI database to find these genes of high similarity. Finally, the evolutionary relationships of genes related to Sb(III) was inferred using the NJ, maximum likelihood (ML), and UPGMA methods in MEGA v7.0 with 1000 bootstraps [5].

### Prediction of mobile genetic elements (MGEs)

GEIs were detected using the web server IslandViewer4 with three prediction methods, including IslandPath-DIMOB, SIGI-HMM, and IslandPick with default parameters [56]. Insertion sequences were predicted and classified using the ISFinder platform against the ISfinder database with default criteria [57]. CRISPR arrays were detected using the CRISPR Finder online server to perform BLAST searches against dbCRISPR (CRISPR database) [58]. PHAST was used to scan prophages by BLASTing against the NCBI and the prophage databases [59].

### Selective pressure analysis and expressivity prediction

The CAI values of selected genes were analyzed using Codon W1.4.2 (http://codonw.sourceforge.net//) and CAI Calculator 2 (http://www.evolvingcode.net/codon/CalculateCAIs.php). The mode and strength of natural selection in protein sequences were estimated by evaluating the ratio of nonsynonymous (*dN*) to synonymous (*dS*) nucleotide substitutions rates using the online software Datamonkey, and the HyPhy package with Single-Likelihood Ancestor Counting method was used to detect the selection sites [60].

### Real-time quantitative PCR (RT-qPCR)

When *Bacillus* sp. S3 growth reached middle exponential phase, culture aliquots were amended with 0, 100, 200 and 300 μM of Sb(III), respectively. The culture aliquots were withdrawn at different time intervals (0.5, 1, 2 and 4 h) and cells were harvested by centrifugation at 4000×*g* for 10 min at 4 °C. After quick freezing in liquid nitrogen, the total RNA of each sample was isolated and purified using the E.Z.B.A Bacterial RNA Kit (Omega, Bio-Tek, USA) according to the manufacturer’s instructions. The concentration of RNA samples was measured at the A_260/280_ ratio using a NanoDrop ND-1000 Spectrophotometer (BioTek Instruments, Inc., Vermont, USA) and the integrity of the RNA samples was determined by 1.0% agarose gel electrophoresis. First-strand cDNA was synthesized with 2 μg of total RNA in a 20 μL total reaction volume using the 5 × HiScriptII qRT SuperMixII (Vazyme Biotech Co., Ltd., China). RT-qPCR analysis was performed using iCycler iQ Real-time PCR detection system (Bio-Rad Laboratories, Inc., Hercules, USA) with a 20 μL reaction volume and using 2 × ChamQ™ Universal SYBR qPCR Master Mix (Vazyme Biotech Co., Ltd., China). Primers were designed using Primer Premier 5 software [19]. Gene expression was calculated by 2^-∆∆Ct^ method as follows: 2^-∆∆Ct^ = 2^-[(CtA-CtB) treated- (CtA-CtB) control]^, where A denotes target gene and B denotes control gene [22].

### Statistical analysis

All experiments were performed in triplicate and results were expressed as mean ± standard deviation (SD). Statistical analyses were carried out by one-way ANOVA with *post-hoc* test-Tukey’s test (*p* < 0.05) in SPSS version 21.0 (SPSS Inc., Chicago, IL, USA).

## Supplementary information

**Additional file 1: Figure S1.** Energy dispersive X-ray spectroscopy (EDS) analysis of *Bacillus* sp. S3 after the different heavy metal ions exposure: (A) Sb(III); (B) As (III); (C) Cd (II); (D) Cr (VI); (E) Pb (II); (F) Cu (II). **Figure S2.** COG classification statistics of the *Bacillus* sp. S3 genome annotation. **Figure S3.** GO classification statistics of the *Bacillus* sp. S3 genome annotation. WEGO was used to produce the graph. **Figure S4.** KEGG classification statistics of the *Bacillus* sp. S3 genome annotation. **Figure S5.** Distribution of CAZymes in *Bacillus* sp. S3. **Figure S6.** Gene contents of the intact prophages in *Bacillus* sp. S3 predicted by PHAST. **Figure S7.** Comparison of G + C contents of these functional genes with those of the average of the entire genomes. (A) *arsB_1*; (B) *arsB_2*; (C) *arsB_3*; (D)*arsC*. **Figure S8.** Neighbor-joining phylogenetic tree of concatenated AioB protein sequences derived from *Bacillus* sp. S3 and other representative species. *Bacillus* sp. S3 was marked in red blot. **Figure S9.** Maximum likelihood phylogenetic tree of concatenated AioB protein sequences derived from *Bacillus* sp. S3 and other representative species. *Bacillus* sp. S3 was marked in red blot. **Figure S10.** Phylogenetic tree analysis based on concatenated AioB protein sequences using UPGMA method under *p*-distance model. Bootstrap values were indicated at each node based on a total of 1000 bootstrap replicates. *Bacillus* sp. S3 was marked in red blot. **Figure S11.** Neighbor-joining phylogenetic tree of concatenated ArsB protein sequences derived from *Bacillus* sp. S3 and other representative species. *Bacillus* sp. S3 was marked in red blot. **Figure S12.** Maximum likelihood phylogenetic tree of concatenated ArsB protein sequences derived from *Bacillus* sp. S3 and other representative species. *Bacillus* sp. S3 was marked in red blot. **Figure S13.** Phylogenetic tree analysis based on concatenated ArsB protein sequences using UPGMA method under *p*-distance model. Bootstrap values were indicated at each node based on a total of 1000 bootstrap replicates. *Bacillus* sp. S3 was marked in red blot. **Figure S14.** Neighbor-joining phylogenetic tree of concatenated ArsC protein sequences derived from *Bacillus* sp. S3 and other representative species. *Bacillus* sp. S3 was marked in red blot. **Figure S15.** Maximum likelihood phylogenetic tree of concatenated ArsC protein sequences derived from *Bacillus* sp. S3 and other representative species. *Bacillus* sp. S3 was marked in red blot. **Figure S16.** Phylogenetic tree analysis based on concatenated ArsC protein sequences using UPGMA method under *p*-distance model. Bootstrap values were indicated at each node based on a total of 1000 bootstrap replicates. *Bacillus* sp. S3 was marked in red blot. **Table S1.** COG functional categories of *Bacillus* sp. S3. **Table S2.** GO categories of *Bacillus* sp. S3. **Table S3.** KEGG categories of *Bacillus* sp. S3. **Table S4.** Digital DNA-DNA hybridization (dDDH) values between *Bacillus* sp. S3 and other *Bacillus* genomes. Formula I, II and III represented different methods used by GGDC to calculate the similarities. **Table S5.** Mobile genetic elements predicted in *Bacillus* genomes by different methods. **Table S6.** Primers used in RT-qPCR. **Table S7.** The quality control of clean data. **Table S8.** The statistical information and of clean data. **Table S9.** The statistical information of genome sequencing and assembling procedures.

## Data Availability

The genome and plasmid sequences of *Bacillus* sp. S3 were deposited in the NCBI database under the accession numbers CP039727.1 and CP039728.1, respectively. All sequences involved in this study are available from the NCBI database.

## Abbreviations

ANI: Average nucleotide identity
BLAST: Basic local alignment search tool
CAI: Codon adaptation index
CDSs: Protein-coding genes
dDDH: digital DNA-DNA hybridization
GEIs: Genomic island
IS: Insertion sequences
LCB: Locally collinear block
MGE: Mobile genetic element
ML: Maximum likelihood
NCBI: National center for biotechnology information
NJ: Neighbor-joining
RT-qPCR: Real-time quantitative polymerase chain reaction
