# Supplementary material for: Complete genome sequencing and comparative genomic analyses of Bacillus sp. S3, a novel hyper Sb(III)-oxidizing bacterium
Source: BMC Microbiol. 2020 May 1;20:106. doi: 10.1186/s12866-020-01737-3 (PMC7193398; doi:10.1186/s12866-020-01737-3)
Supplement: Supplementary file 1 — Additional file 1: Figure S1. Energy dispersive X-ray spectroscopy (EDS) analysis of Bacillus sp. S3 after the different heavy metal ions exposure: (A) Sb(III); (B) As (III); (C) Cd (II); (D) Cr (VI); (E) Pb (II); (F) Cu (II). Figure S2. COG classification statistics of the Bacillus sp. S3 genome annotation. Figure S3. GO classification statistics of the Bacillus sp. S3 genome annotation. WEGO was used to produce the graph. Figure S4. KEGG classification statistics of the Bacillus sp. S3 genome annotation. Figure S5. Distribution of CAZymes in Bacillus sp. S3. Figure S6. Gene contents of the intact prophages in Bacillus sp. S3 predicted by PHAST. Figure S7. Comparison of G + C contents of these functional genes with those of the average of the entire genomes. (A) arsB_1; (B) arsB_2; (C) arsB_3; (D)arsC. Figure S8. Neighbor-joining phylogenetic tree of concatenated AioB protein sequences derived from Bacillus sp. S3 and other representative species. Bacillus sp. S3 was marked in red blot. Figure S9. Maximum likelihood phylogenetic tree of concatenated AioB protein sequences derived from Bacillus sp. S3 and other representative species. Bacillus sp. S3 was marked in red blot. Figure S10. Phylogenetic tree analysis based on concatenated AioB protein sequences using UPGMA method under p-distance model. Bootstrap values were indicated at each node based on a total of 1000 bootstrap replicates. Bacillus sp. S3 was marked in red blot. Figure S11. Neighbor-joining phylogenetic tree of concatenated ArsB protein sequences derived from Bacillus sp. S3 and other representative species. Bacillus sp. S3 was marked in red blot. Figure S12. Maximum likelihood phylogenetic tree of concatenated ArsB protein sequences derived from Bacillus sp. S3 and other representative species. Bacillus sp. S3 was marked in red blot. Figure S13. Phylogenetic tree analysis based on concatenated ArsB protein sequences using UPGMA method under p-distance model. Bootstrap values were indicated at each n [file 12866_2020_1737_MOESM1_ESM.docx]

**Supplementary Information**

**Complete sequencing and comparative genomic analyses of *Bacillus* sp. S3, a novel hyper Sb(III)-oxidizing bacterium**

**Jiaokun Li^1,2^, Tianyuan Gu^1,2^, Liangzhi Li^1,2^, Xueling Wu^1,2^, Li Shen^1,2^, Runlan Yu^1,2^, Yuandong Liu^1,2^, Guanzhou Qiu^1,2^ and Weimin Zeng^1,2*^**

*** Corresponding author**

**Affiliations:**

^1^ School of Minerals Processing and Bioengineering, Central South University, Changsha, 410083, China

^2^ Key Laboratory of Biometallurgy, Ministry of Education, Central South University, Changsha, 410083, China

**Figure S1.** Energy dispersive X-ray spectroscopy (EDS) analysis of *Bacillus* sp. S3 after the different heavy metal ions exposure: (A) Sb(III); (B) As(III); (C) Cd(II); (D) Cr(VI); (E) Pb(II); (F) Cu(II).


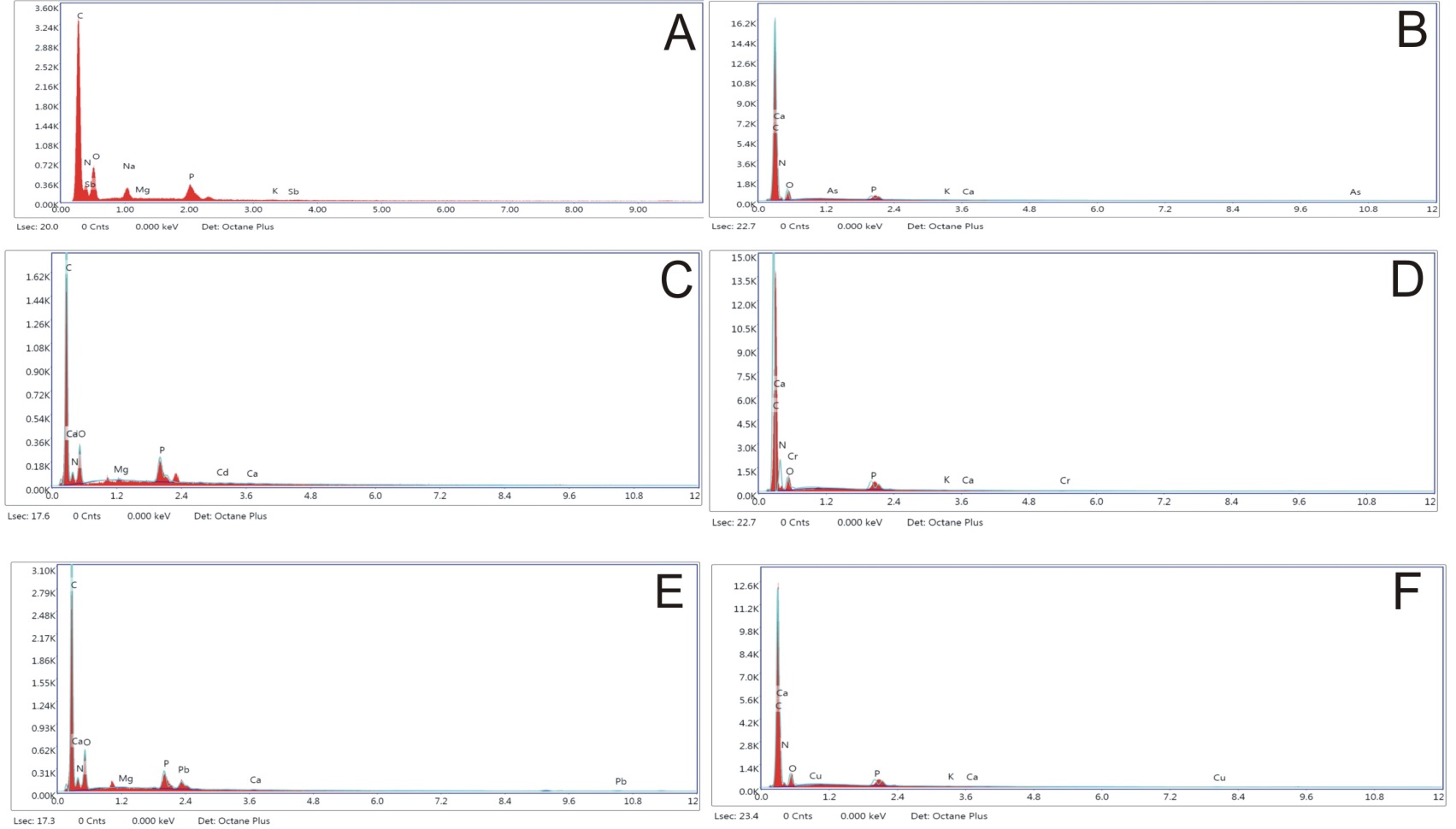


**Figure S2.** COG classification statistics of the *Bacillus* sp. S3 genome annotation.

**
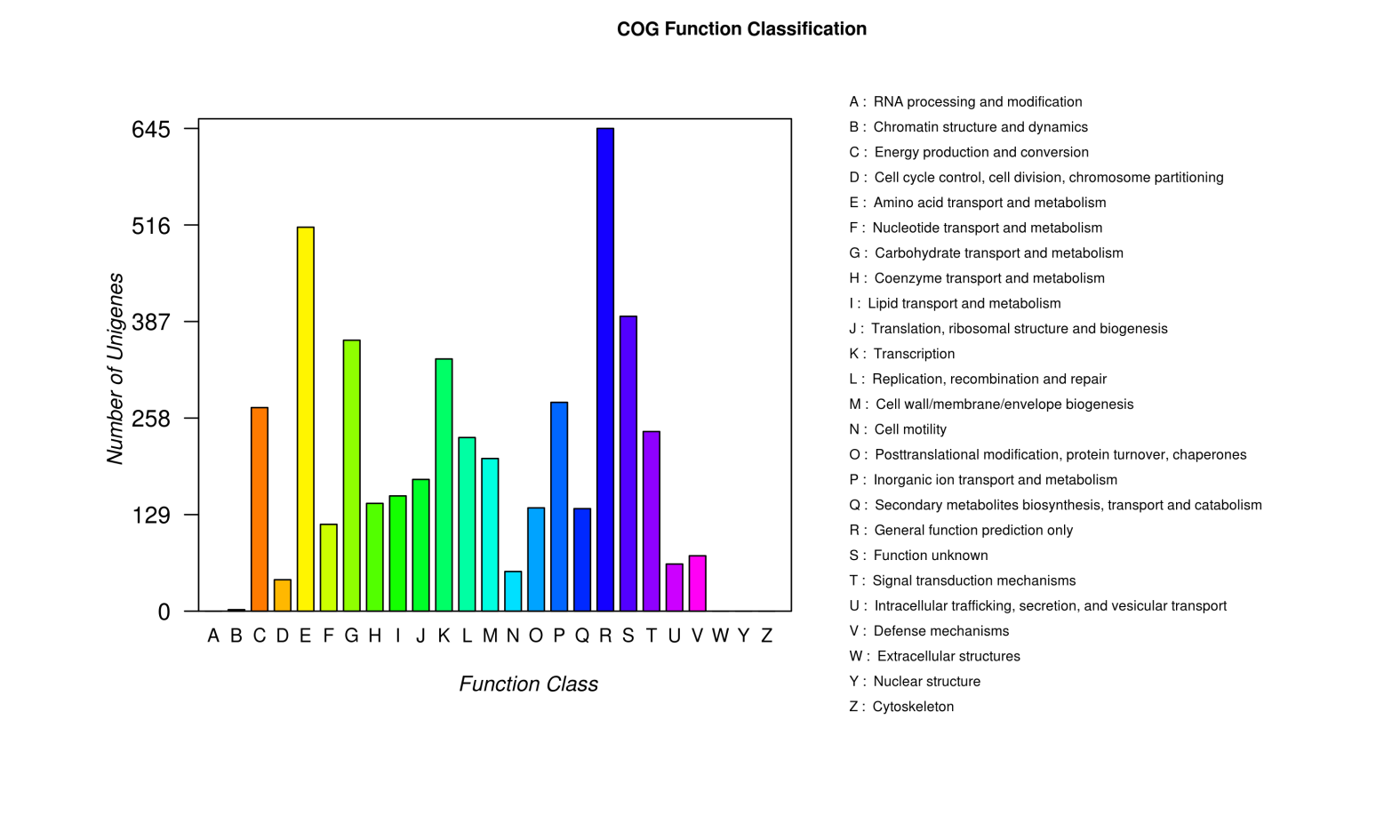
**

**Figure S3.** GO classification statistics of the *Bacillus* sp. S3 genome annotation. WEGO was used to produce the graph.


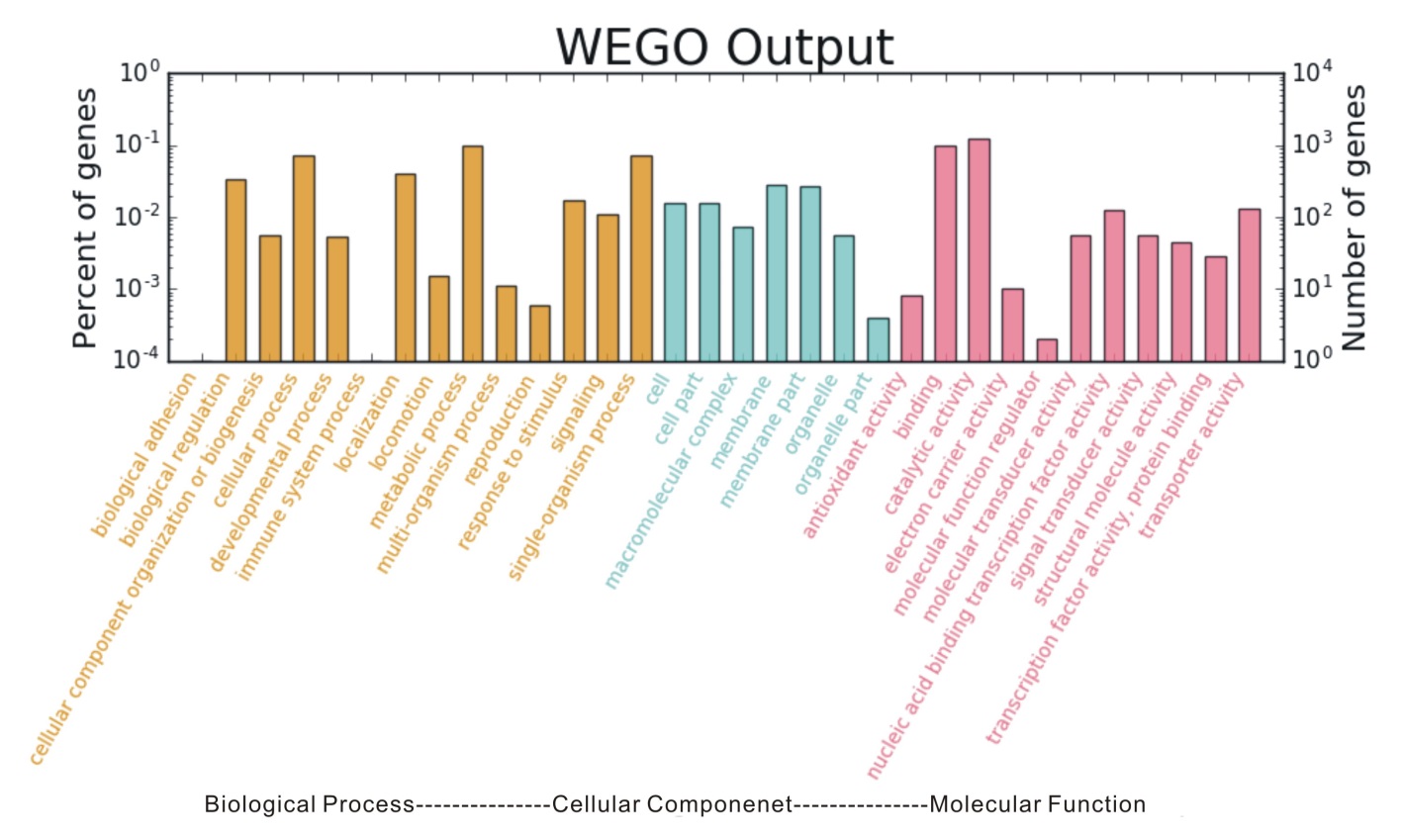


**Figure S4.** KEGG classification statistics of the *Bacillus* sp. S3 genome annotation.


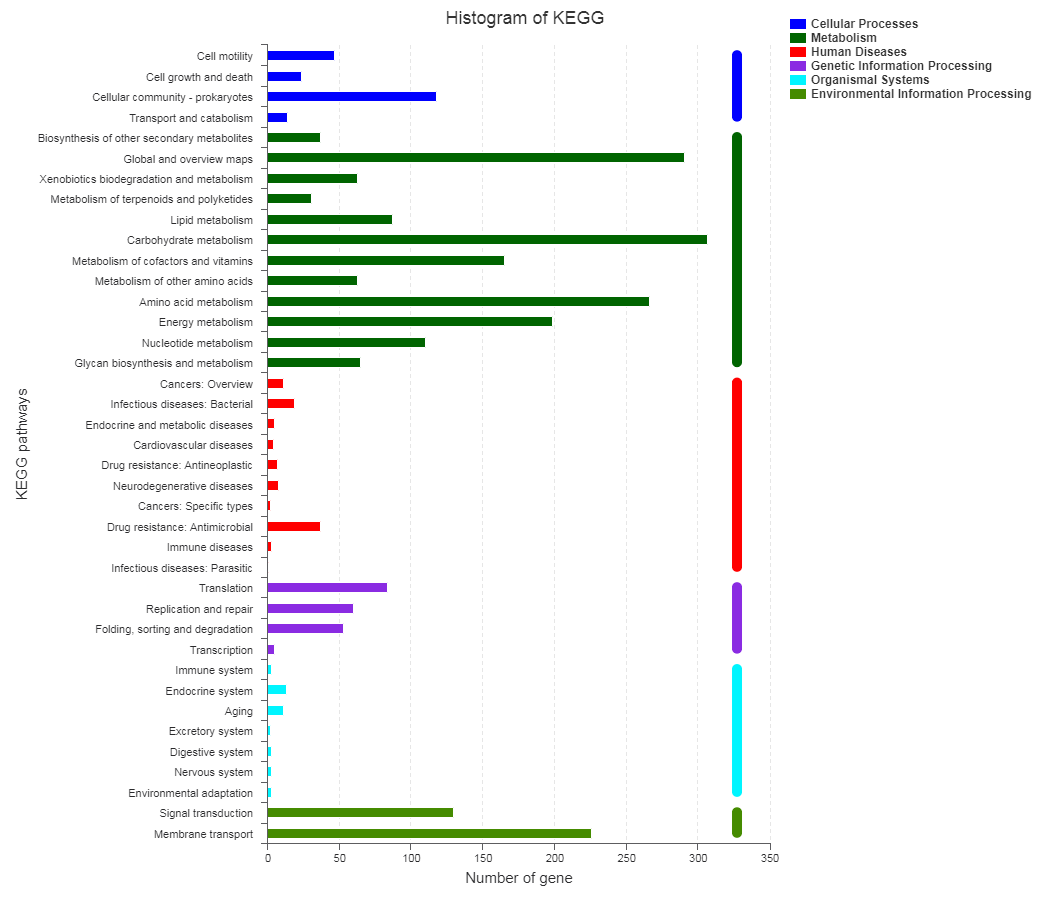


**Figure S5.** Distribution of CAZymes in *Bacillus* sp. S3.


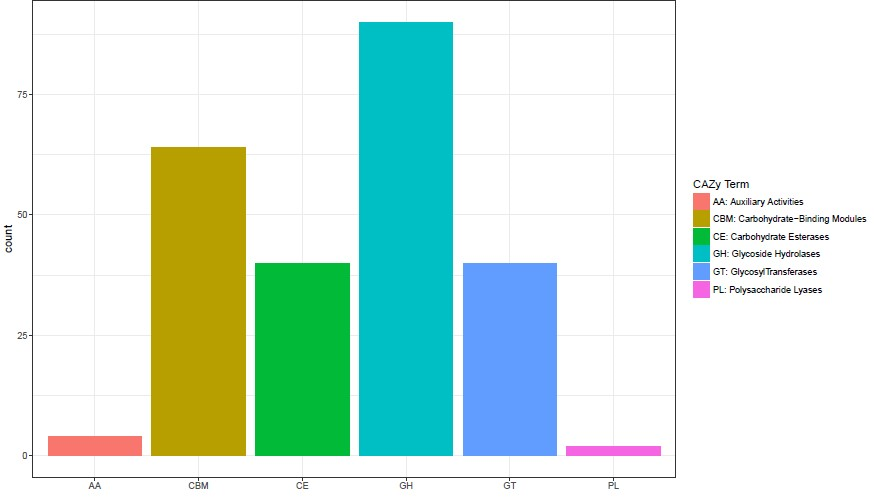


**Figure S6.** Gene contents of the intact prophages in *Bacillus* sp. S3 predicted by PHAST.

**
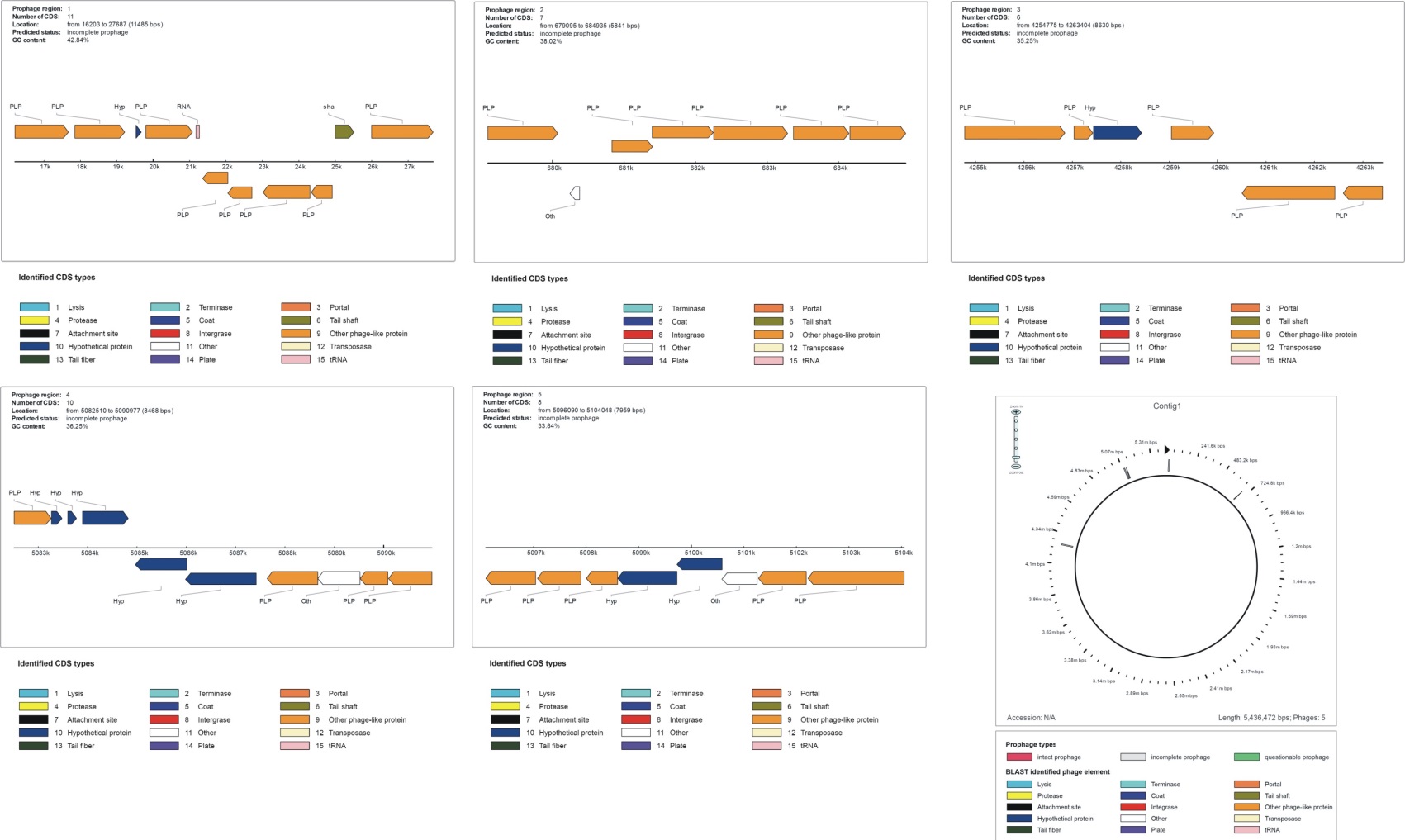
**

**Figure S7.** Comparison of G+C contents of these functional genes with those of the average of the entire genomes. (A) *arsB_1*; (B) *arsB_2*; (C) *arsB_3*; (D)*arsC*.


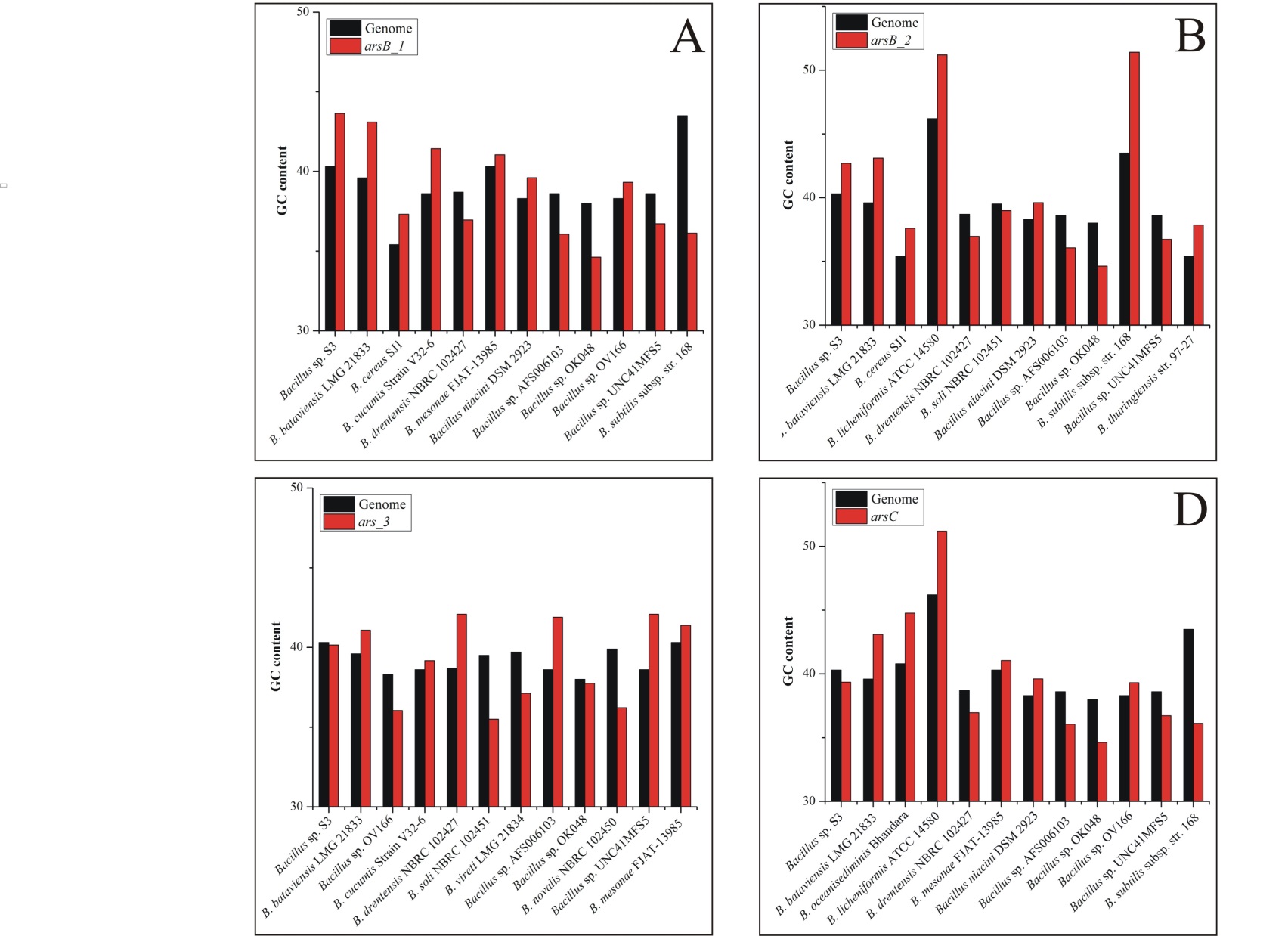


**Figure S8.** Neighbor-joining phylogenetic tree of concatenated AioB protein sequences derived from *Bacillus* sp. S3 and other representative species. *Bacillus* sp. S3 was marked in red blot.

**
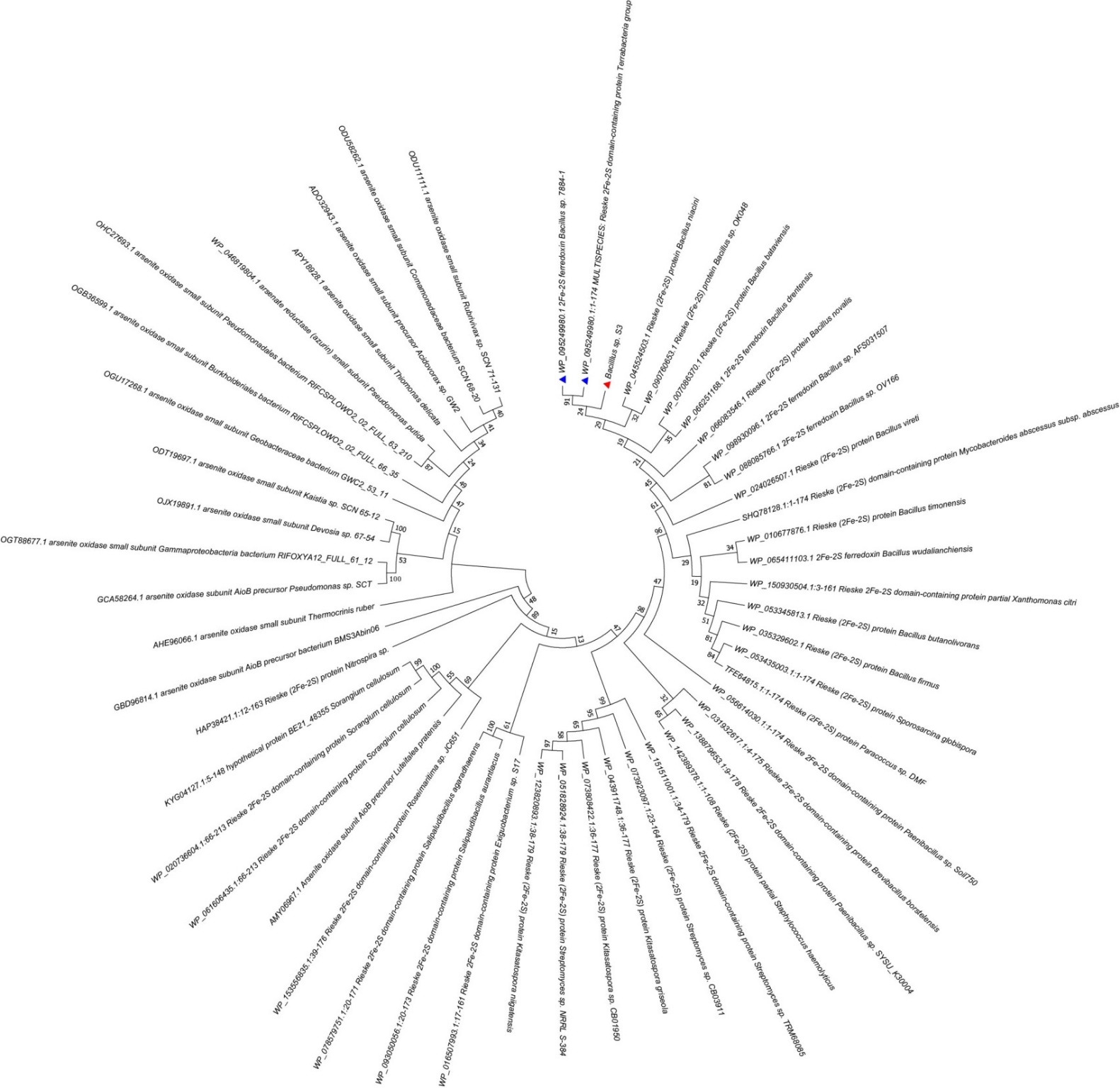
**

**Figure S9.** Maximum likelihood phylogenetic tree of concatenated AioB protein sequences derived from *Bacillus* sp. S3 and other representative species. *Bacillus* sp. S3 was marked in red blot.

**
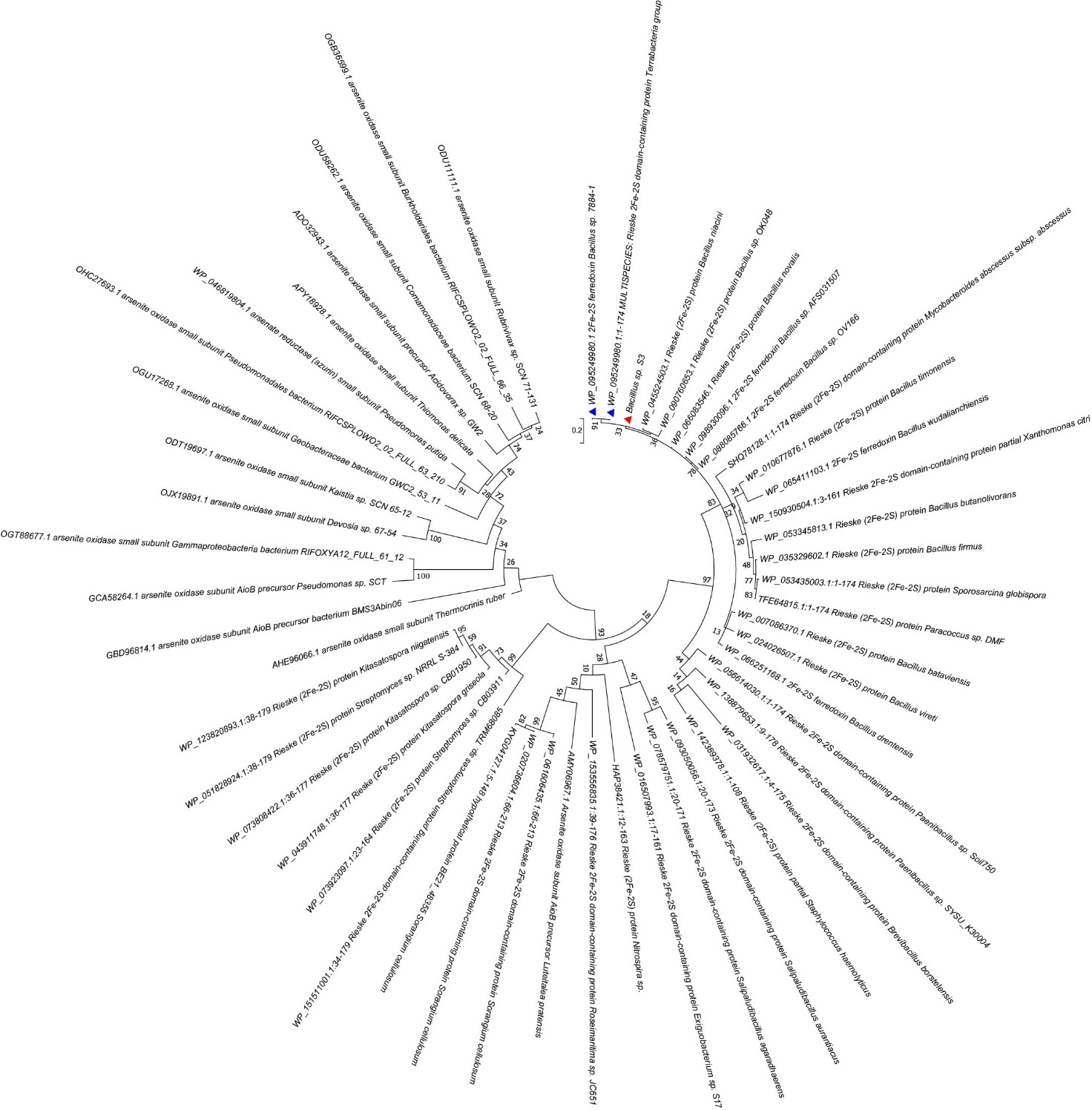
**

**Figure S10.** Phylogenetic tree analysis based on concatenated AioB protein sequences using UPGMA method under *p*-distance model. Bootstrap values were indicated at each node based on a total of 1,000 bootstrap replicates. *Bacillus* sp. S3 was marked in red blot.

**
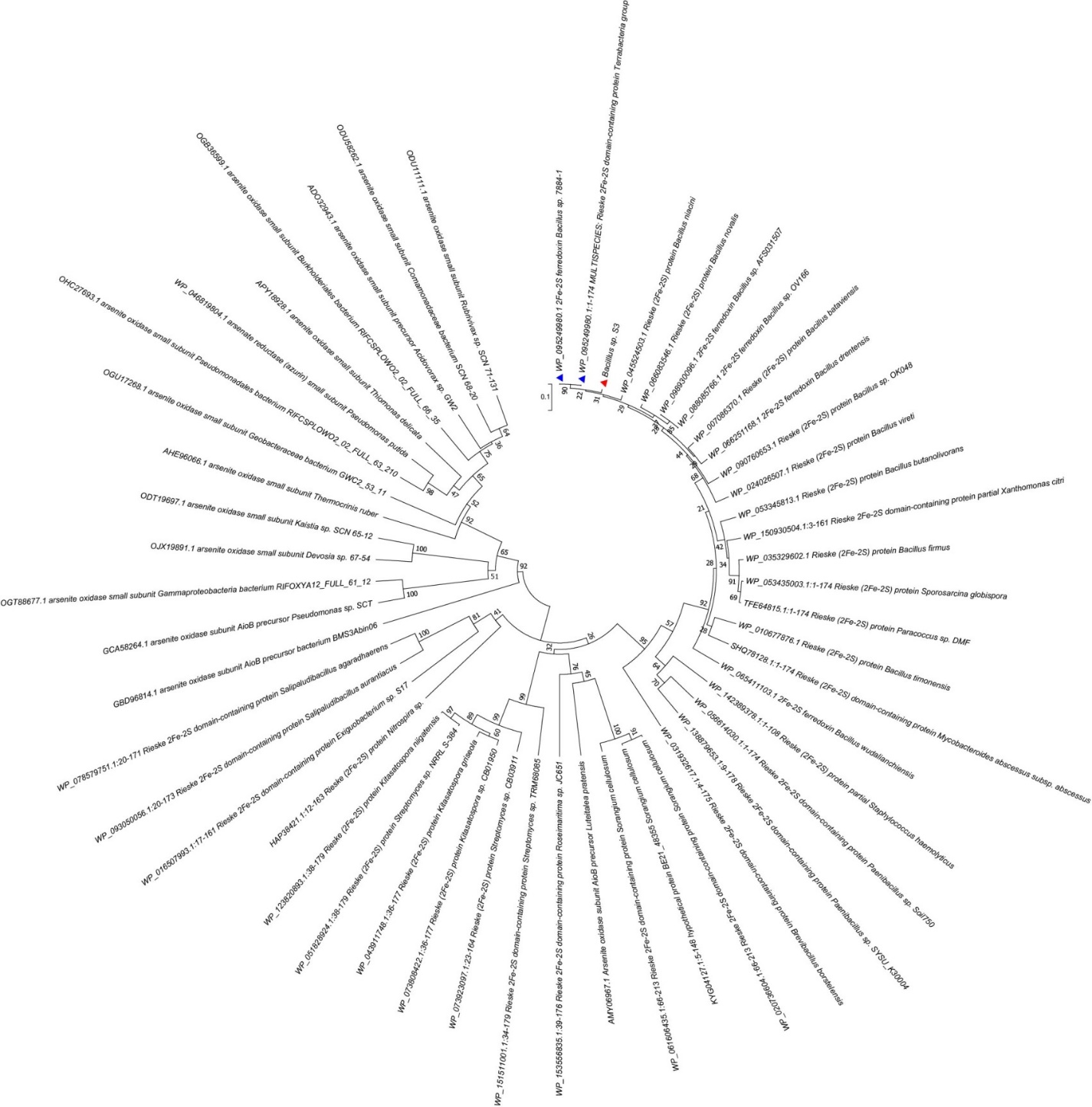
**

**Figure S11.** Neighbor-joining phylogenetic tree of concatenated ArsB protein sequences derived from *Bacillus* sp. S3 and other representative species. *Bacillus* sp. S3 was marked in red blot.


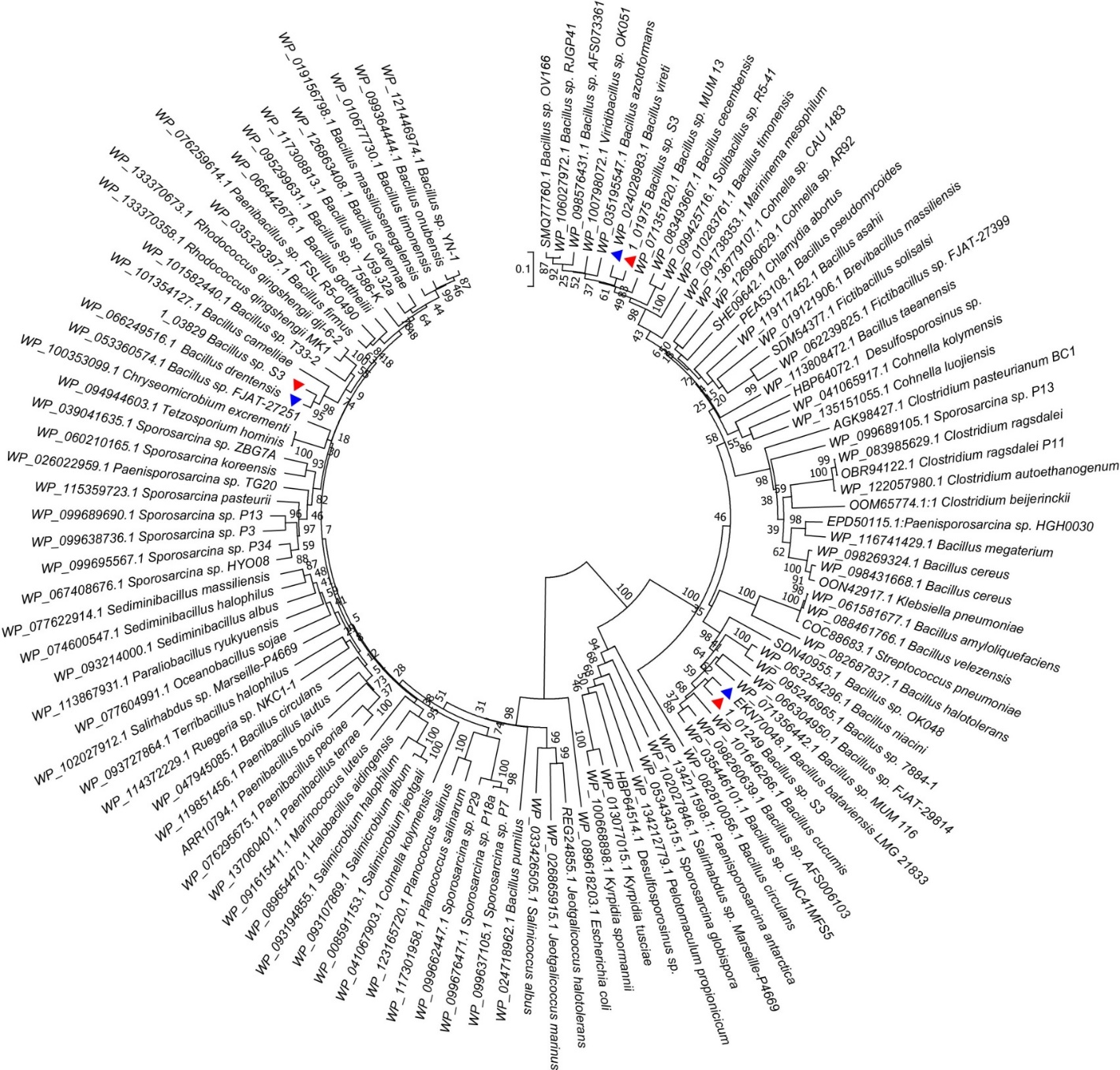


**Figure S12.** Maximum likelihood phylogenetic tree of concatenated ArsB protein sequences derived from *Bacillus* sp. S3 and other representative species. *Bacillus* sp. S3 was marked in red blot.

**
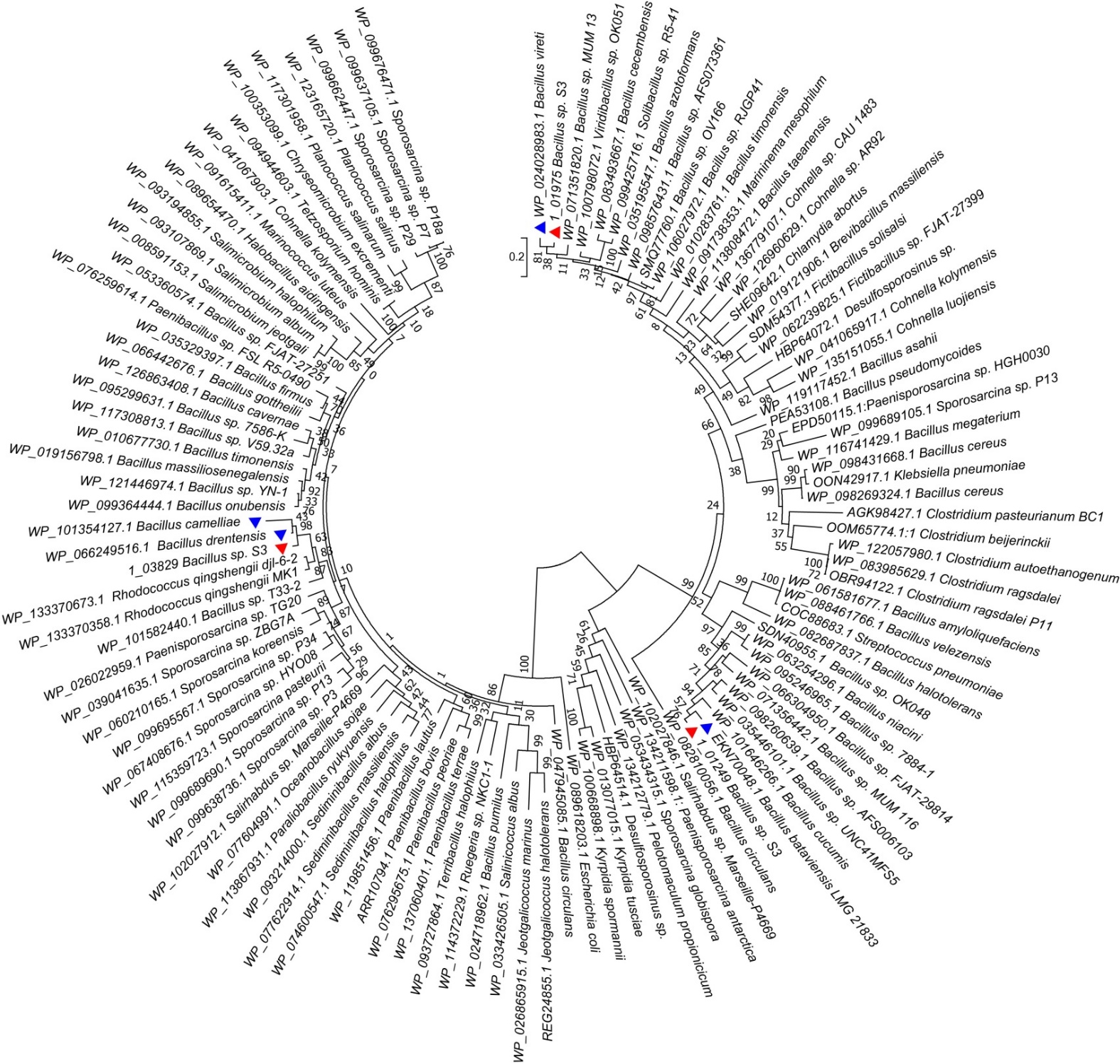
**

**Figure S13.** Phylogenetic tree analysis based on concatenated ArsB protein sequences using UPGMA method under *p*-distance model. Bootstrap values were indicated at each node based on a total of 1,000 bootstrap replicates. *Bacillus* sp. S3 was marked in red blot.

**
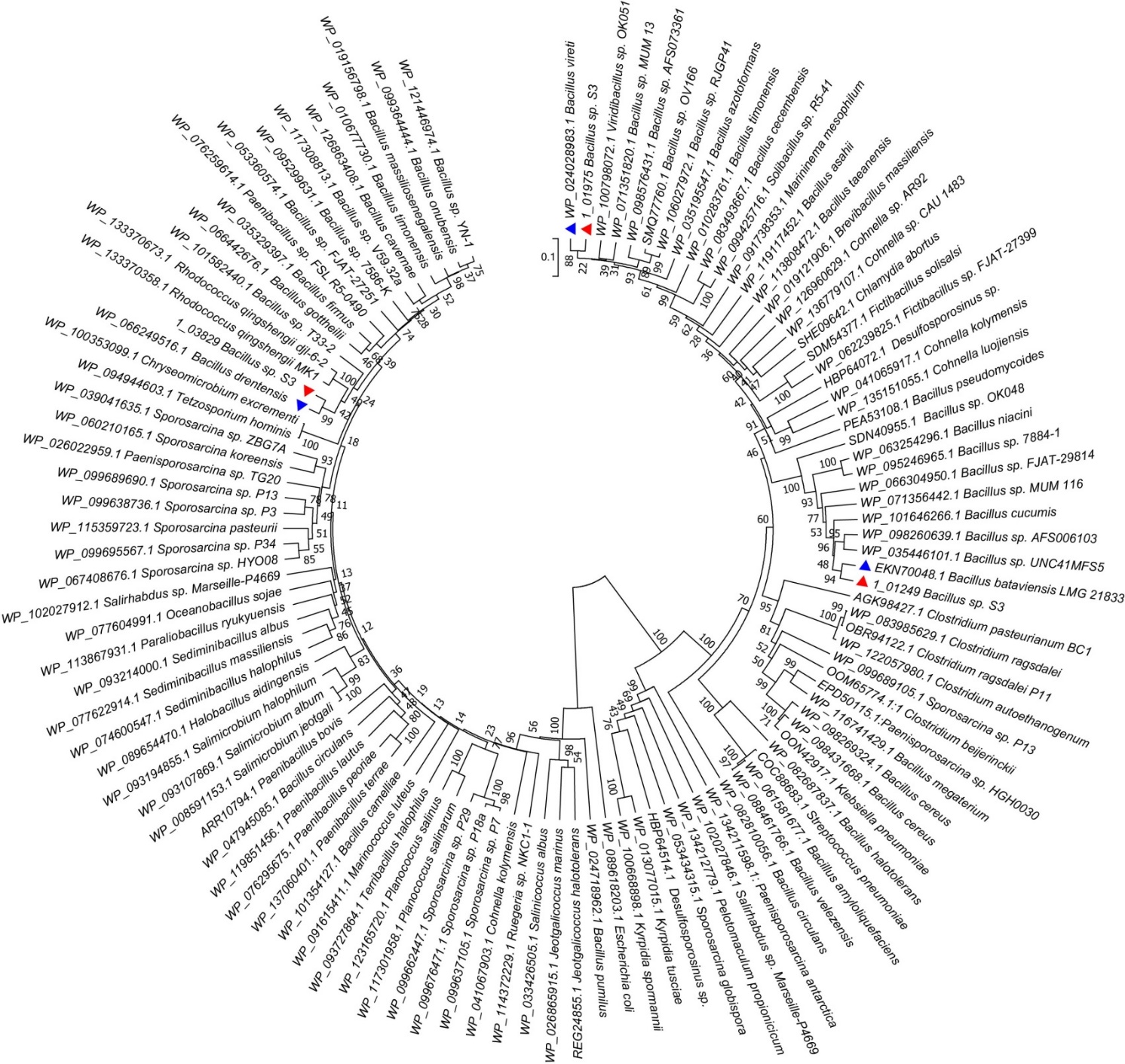
**

**Figure S14.** Neighbor-joining phylogenetic tree of concatenated ArsC protein sequences derived from *Bacillus* sp. S3 and other representative species. *Bacillus* sp. S3 was marked in red blot.

**
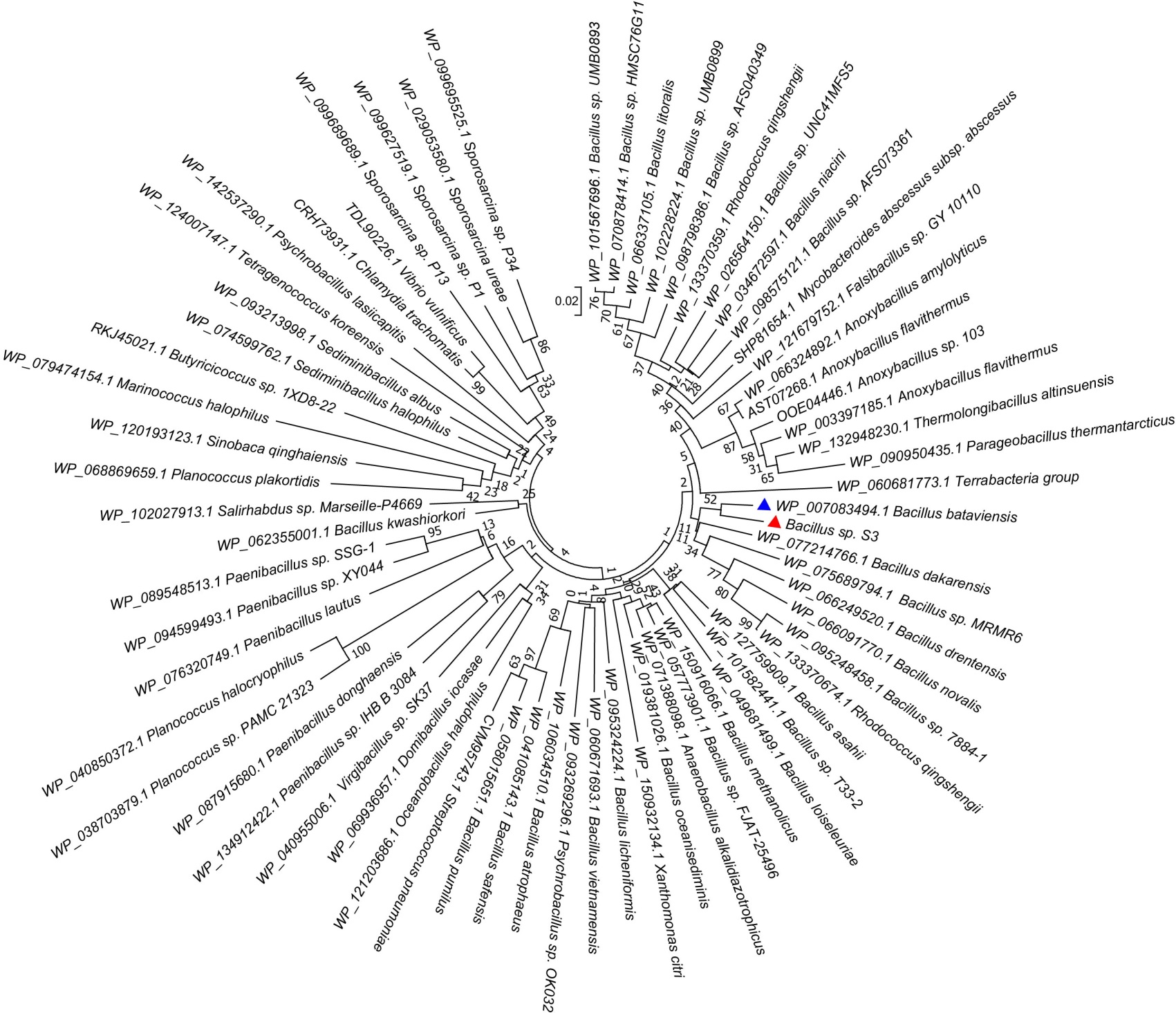
**

**Figure S15.** Maximum likelihood phylogenetic tree of concatenated ArsC protein sequences derived from *Bacillus* sp. S3 and other representative species. *Bacillus* sp. S3 was marked in red blot.

**
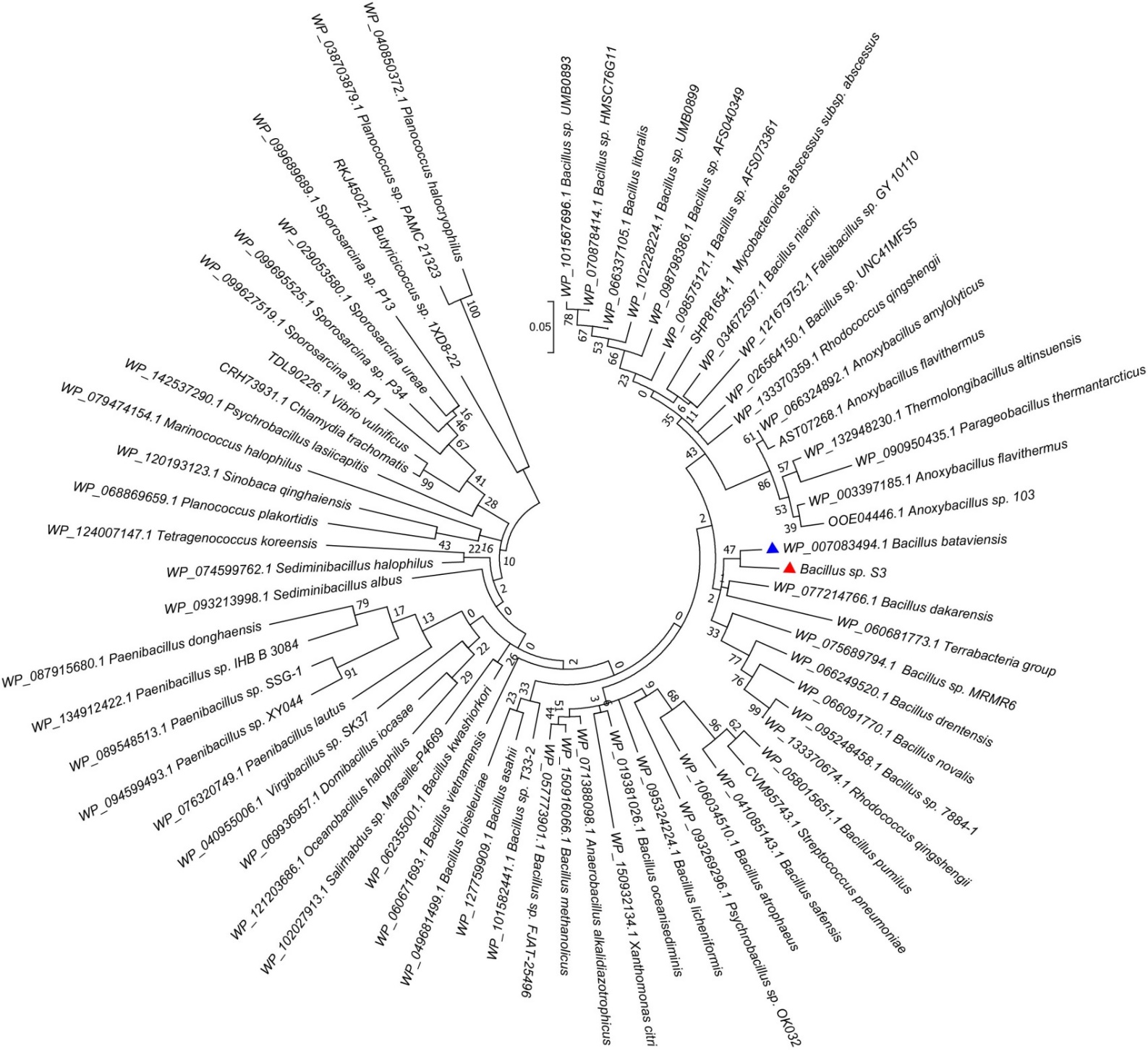
**

**Figure S16.** Phylogenetic tree analysis based on concatenated ArsC protein sequences using UPGMA method under *p*-distance model. Bootstrap values were indicated at each node based on a total of 1,000 bootstrap replicates. *Bacillus* sp. S3 was marked in red blot.

**
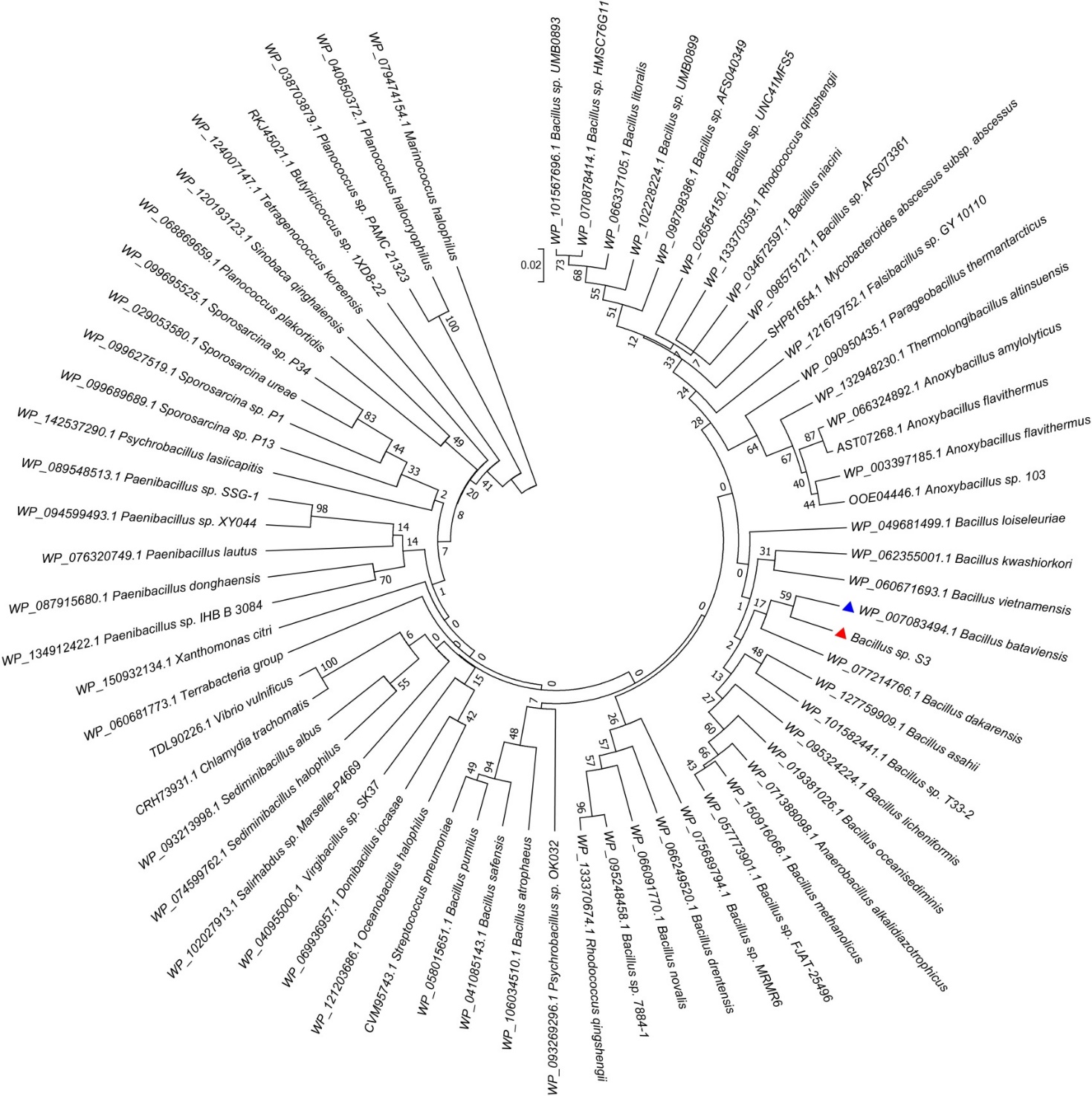
**

**Table S1.** COG functional categories of *Bacillus* sp. S3.

| Category | Type | Function Description | Count | Percent (%) |
| --- | --- | --- | --- | --- |
| Metabolism | C | Energy production and conversion | 233 | 5.33 |
|  | E | Amino acid transport and metabolism | 352 | 8.05 |
|  | F | Nucleotide transport and metabolism | 93 | 2.13 |
|  | G | Carbohydrate transport and metabolism | 269 | 6.15 |
|  | H | Coenzyme transport and metabolism | 107 | 2.45 |
|  | I | Lipid transport and metabolism | 93 | 2.13 |
|  | P | Inorganic ion transport and metabolism | 193 | 4.42 |
|  | Q | Secondary metabolites biosynthesis, transport and catabolism | 44 | 1.01 |
| Cellular processes and signaling | D | Cell cycle control, Cell division, chromosome partitioning | 42 | 0.96 |
|  | M | Cell wall/membrane biogenesis | 179 | 4.10 |
|  | N | Cell motility | 19 | 0.43 |
|  | O | Posttranslational modification, protein turnover, chaperones | 114 | 2.61 |
|  | T | Signal transduction mechanisms | 141 | 3.23 |
|  | U | Intracellular trafficking and secretion | 33 | 0.75 |
|  | V | Defense mechanisms | 74 | 1.69 |
|  | W | Extracellular structures | 0 | 0.00 |
|  | Y | Nuclear structure | 0 | 0.00 |
|  | Z | Cytoskeleton | 0 | 0.00 |
| Information storage and processing | J | Translation, ribosomal structure and biogenesis | 165 | 3.77 |
|  | A | RNA processing and modification | 0 | 0.00 |
|  | B | Chromatin structure and dynamics | 0 | 0.00 |
|  | K | Transcription | 221 | 5.06 |
|  | L | Replication, recombination and repair | 201 | 4.60 |
| Poorly characterized | R | General function prediction only | 450 | 10.30 |
|  | S | Function unknown | 393 | 8.99 |
|  | - | Not in COGs | 955 | 21.85 |

**Table S2.** GO categories of *Bacillus* sp. S3.

| Category | Function Description | Count |
| --- | --- | --- |
| Molecular function | Transporter activity | 134 |
|  | Signal transducer activity | 57 |
|  | Electron carrier activity | 10 |
|  | Nucleic acid binding transcription factor activity | 124 |
|  | Transcription factor activity, protein binding | 29 |
|  | Molecular function regulator | 2 |
|  | Antioxidant activity | 8 |
|  | Molecular transducer activity | 56 |
|  | Binding | 1,002 |
|  | Structural molecule activity | 45 |
|  | Catalytic activity | 1,248 |
| Cellular component | Cell | 159 |
|  | Macromolecular complex | 75 |
|  | Cell part | 159 |
|  | Membrane | 283 |
|  | Organelle part | 4 |
|  | Organelle | 55 |
|  | Membrane part | 266 |
| Biological process | Locomotion | 15 |
|  | Cellular component organization or biogenesis | 57 |
|  | Metabolic process | 980 |
|  | Biological regulation | 341 |
|  | Biological adhesion | 1 |
|  | Multi-organism process | 11 |
|  | Reproduction | 6 |
|  | Cellular process | 718 |
|  | Response to stimulus | 170 |
|  | Single-organism process | 735 |
|  | Immune system process | 1 |
|  | Localization | 411 |
|  | Signaling | 110 |
|  | Developmental process | 54 |

**Table S3.** KEGG categories of *Bacillus* sp. S3.

| No. | Count | Function Description |
| --- | --- | --- |
| ko01100 | 637 | Metabolic pathways |
| ko01110 | 285 | Biosynthesis of secondary metabolites |
| ko01120 | 215 | Microbial metabolism in diverse environments |
| ko01200 | 135 | Carbon metabolism |
| ko01230 | 134 | Biosynthesis of amino acids |
| ko02010 | 122 | ABC transporters |
| ko02020 | 87 | Two-component system |
| ko00230 | 61 | Purine metabolism |
| ko00720 | 52 | Carbon fixation pathways in prokaryotes |
| ko00330 | 49 | Arginine and proline metabolism |
| ko00190 | 48 | Oxidative phosphorylation |
| ko00260 | 47 | Glycine, serine and threonine metabolism |
| ko00620 | 47 | Pyruvate metabolism |
| ko00680 | 46 | Methane metabolism |
| ko00010 | 45 | Glycolysis/Gluconeogenesis |
| ko00240 | 45 | Pyrimidine metabolism |
| ko03010 | 45 | Ribosome |
| ko00520 | 45 | Amino sugar and nucleotide sugar metabolism |
| ko00650 | 43 | Butanoate metabolism |
| ko01212 | 41 | Fatty acid metabolism |
| ko00640 | 40 | Propanoate metabolism |
| ko00630 | 36 | Glyoxylate and dicarboxylate metabolism |
| ko00020 | 36 | Citrate cycle (TCA cycle) |
| ko00250 | 34 | Alanine, aspartate and glutamate metabolism |
| ko00270 | 33 | Cysteine and methionine metabolism |
| ko00030 | 33 | Pentose phosphate pathway |
| ko00500 | 32 | Starch and sucrose metabolism |
| ko02060 | 31 | Phosphotransferase system (PTS) |
| ko02040 | 29 | Flagellar assembly |
| ko00910 | 29 | Nitrogen metabolism |
| ko00280 | 28 | Valine, leucine and isoleucine degradation |
| ko00970 | 26 | Aminoacyl-tRNA biosynthesis |
| ko00051 | 26 | Fructose and mannose metabolism |
| ko00061 | 25 | Fatty acid biosynthesis |
| ko01210 | 25 | 2-Oxocarboxylic acid metabolism |
| ko00400 | 24 | Phenylalanine, tyrosine and tryptophan biosynthesis |
| ko00564 | 24 | Glycerophospholipid metabolism |
| ko00052 | 24 | Galactose metabolism |
| ko00340 | 22 | Histidine metabolism |
| ko02030 | 22 | Bacterial chemotaxis |
| ko00860 | 22 | Porphyrin and chlorophyll metabolism |
| ko00040 | 20 | Pentose and glucuronate interconversions |
| ko00071 | 19 | Fatty acid degradation |
| ko03060 | 19 | Protein export |
| ko00360 | 18 | Phenylalanine metabolism |
| ko00710 | 18 | Carbon fixation in photosynthetic organisms |
| ko00300 | 17 | Lysine biosynthesis |
| ko03430 | 17 | Mismatch repair |
| ko00780 | 16 | Biotin metabolism |
| ko00380 | 16 | Tryptophan metabolism |
| ko00790 | 16 | Folate biosynthesis |
| ko00550 | 16 | Peptidoglycan biosynthesis |
| ko00450 | 15 | Selenocompound metabolism |
| ko00900 | 15 | Terpenoid backbone biosynthesis |
| ko03070 | 14 | Bacterial secretion system |
| ko01040 | 14 | Biosynthesis of unsaturated fatty acids |
| ko00290 | 14 | Valine, leucine and isoleucine biosynthesis |
| ko03440 | 14 | Homologous recombination |
| ko00362 | 14 | Benzoate degradation |
| ko00310 | 14 | Lysine degradation |
| ko00770 | 14 | Pantothenate and CoA biosynthesis |
| ko00633 | 13 | Nitrotoluene degradation |
| ko00760 | 13 | Nicotinate and nicotinamide metabolism |
| ko03030 | 13 | DNA replication |
| ko00920 | 13 | Sulfur metabolism |
| ko00350 | 13 | Tyrosine metabolism |
| ko00670 | 13 | One carbon pool by folate |
| ko00730 | 12 | Thiamine metabolism |
| ko04122 | 12 | Sulfur relay system |
| ko04112 | 12 | Cell cycle-Caulobacter |
| ko03018 | 12 | RNA degradation |
| ko00410 | 12 | Beta-Alanine metabolism |
| ko03420 | 12 | Nucleotide excision repair |
| ko04146 | 11 | Peroxisome |
| ko03410 | 11 | Base excision repair |
| ko00430 | 11 | Taurine and hypotaurine metabolism |
| ko01220 | 10 | Degradation of aromatic compounds |
| ko00660 | 10 | C5-Branched dibasic acid metabolism |
| ko00561 | 10 | Glycerolipid metabolism |
| ko00627 | 10 | Aminobenzoate degradation |
| ko00195 | 9 | Photosynthesis |
| ko00562 | 9 | Inositol phosphate metabolism |
| ko00521 | 9 | Streptomycin biosynthesis |
| ko00511 | 8 | Other glycan degradation |
| ko00460 | 8 | Cyanoamino acid metabolism |
| ko05150 | 8 | Staphylococcus aureus infection |
| ko00130 | 8 | Ubiquinone and other terpenoid-quinone biosynthesis |
| ko04066 | 7 | HIF-1 signaling pathway |
| ko00072 | 7 | Synthesis and degradation of ketone bodies |
| ko00480 | 7 | Glutathione metabolism |
| ko00531 | 7 | Glycosaminoglycan degradation |
| ko00983 | 7 | Drug metabolism-other enzymes |
| ko00625 | 7 | Chloroalkane and chloroalkene degradation |
| ko00281 | 6 | Geraniol degradation |
| ko00401 | 6 | Novobiocin biosynthesis |
| ko00253 | 6 | Tetracycline biosynthesis |
| ko00622 | 6 | Xylene degradation |
| ko00740 | 6 | Riboflavin metabolism |
| ko00523 | 6 | Polyketide sugar unit biosynthesis |
| ko00473 | 5 | D-Alanine metabolism |
| ko05111 | 5 | Vibrio cholerae pathogenic cycle |
| ko00750 | 5 | Vitamin B6 metabolism |
| ko00960 | 5 | Tropane, piperidine and pyridine alkaloid biosynthesis |
| ko03320 | 5 | PPAR signaling pathway |
| ko00621 | 5 | Dioxin degradation |
| ko00903 | 5 | Limonene and pinene degradation |
| ko04141 | 4 | Protein processing in endoplasmic reticulum |
| ko00643 | 4 | Styrene degradation |
| ko03020 | 4 | RNA polymerase |
| ko00440 | 4 | Phosphonate and phosphinate metabolism |
| ko00471 | 4 | D-Glutamine and D-glutamate metabolism |
| ko00626 | 4 | Naphthalene degradation |
| ko00053 | 4 | Ascorbate and aldarate metabolism |
| ko04626 | 4 | Plant-pathogen interaction |
| ko05014 | 4 | Amyotrophic lateral sclerosis (ALS) |
| ko05016 | 4 | Huntington's disease |
| ko05134 | 3 | Legionellosis |
| ko05132 | 3 | Salmonella infection |
| ko00785 | 3 | Lipoic acid metabolism |
| ko04920 | 3 | Adipocytokine signaling pathway |
| ko00930 | 3 | Caprolactam degradation |
| ko00600 | 3 | Sphingolipid metabolism |
| ko04070 | 3 | Phosphatidylinositol signaling system |
| ko00940 | 3 | Phenylpropanoid biosynthesis |
| ko04724 | 3 | Glutamatergic synapse |
| ko04727 | 3 | GABAergic synapse |
| ko05340 | 3 | Primary immunodeficiency |
| ko05152 | 3 | Tuberculosis |
| ko05120 | 3 | Epithelial cell signaling in Helicobacter pylori infection |
| ko00623 | 3 | Toluene degradation |
| ko04964 | 2 | Proximal tubule bicarbonate reclamation |
| ko04910 | 2 | Insulin signaling pathway |
| ko05020 | 2 | Prion diseases |
| ko00472 | 2 | D-Arginine and D-ornithine metabolism |
| ko05010 | 2 | Alzheimer's disease |
| ko04011 | 2 | MAPK signaling pathway-yeast |
| ko00909 | 2 | Sesquiterpenoid and triterpenoid biosynthesis |
| ko04973 | 2 | Carbohydrate digestion and absorption |
| ko04978 | 2 | Mineral absorption |
| ko00950 | 2 | Isoquinoline alkaloid biosynthesis |
| ko00590 | 2 | Arachidonic acid metabolism |
| ko03013 | 2 | RNA transport |
| ko00524 | 2 | Butirosin and neomycin biosynthesis |
| ko00791 | 2 | Atrazine degradation |
| ko04142 | 1 | Lysosome |
| ko00510 | 1 | N-Glycan biosynthesis |
| ko01051 | 1 | Biosynthesis of ansamycins |
| ko00941 | 1 | Flavonoid biosynthesis |
| ko05215 | 1 | Prostate cancer |
| ko00642 | 1 | Ethylbenzene degradation |
| ko03008 | 1 | Ribosome biogenesis in eukaryotes |
| ko05204 | 1 | Chemical carcinogenesis |
| ko00603 | 1 | Glycosphingolipid biosynthesis-globo series |
| ko00604 | 1 | Glycosphingolipid biosynthesis-ganglio series |
| ko04918 | 1 | Thyroid hormone synthesis |
| ko04917 | 1 | Prolactin signaling pathway |
| ko04915 | 1 | Estrogen signaling pathway |
| ko04914 | 1 | Progesterone-mediated oocyte maturation |
| ko05205 | 1 | Proteoglycans in cancer |
| ko05206 | 1 | MicroRNAs in cancer |
| ko05200 | 1 | Pathways in cancer |
| ko05203 | 1 | Viral carcinogenesis |
| ko05146 | 1 | Amoebiasis |
| ko01053 | 1 | Biosynthesis of siderophore group nonribosomal peptides |
| ko00943 | 1 | Isoflavonoid biosynthesis |
| ko00361 | 1 | Chlorocyclohexane and chlorobenzene degradation |
| ko00363 | 1 | Bisphenol degradation |
| ko00980 | 1 | Metabolism of xenobiotics by cytochrome P450 |
| ko00982 | 1 | Drug metabolism-cytochrome P450 |
| ko04151 | 1 | PI3K-Akt signaling pathway |
| ko00312 | 1 | Beta-Lactam resistance |
| ko04712 | 1 | Circadian rhythm-plant |
| ko00592 | 1 | Alpha-Linolenic acid metabolism |
| ko00591 | 1 | Linoleic acid metabolism |
| ko00624 | 1 | Polycyclic aromatic hydrocarbon degradation |
| ko04612 | 1 | Antigen processing and presentation |
| ko04621 | 1 | NOD-like receptor signaling pathway |
| ko00830 | 1 | Retinol metabolism |
| ko00908 | 1 | Zeatin biosynthesis |
| ko04930 | 1 | Type II diabetes mellitus |

**Table S4.** Digital DNA-DNA hybridization (dDDH) values between *Bacillus* sp. S3 and other *Bacillus* genomes. Formula I, II and III represented different methods used by GGDC to calculate the similarities.

| Query genome | Reference genome | Formula 1 | | | Formula 2 | | | Formula 3 | | |
| --- | --- | --- | --- | --- | --- | --- | --- | --- | --- | --- |
|  |  | DDH (%) | Model C.I. (%) | Distance | DDH (%) | Model C.I. (%) | Distance | DDH (%) | Model C.I. (%) | Distance |
| *Bacillus* sp. S3 | *B. bataviensis* LMG 21833 | 30.2 | 26.8-33.8 | 0.5105 | 25.9 | 23.6-28.4 | 0.5105 | 28.1 | 25.2-31.2 | 0.5924 |
| *Bacillus* sp. S3 | *B. vireti* LMG 21834 | 21.5 | 18.3-25.1 | 0.6733 | 23.3 | 21-25.7 | 0.1880 | 20.8 | 18-23.8 | 0.7347 |
| *Bacillus* sp. S3 | *B. vireti* DSM 15602 | 21.7 | 18.5-25.3 | 0.6687 | 23.4 | 21.1-25.8 | 0.1872 | 21 | 18.2-24 | 0.7307 |
| *Bacillus* sp. S3 | *B. soli* NBRC 102451 | 21.4 | 18.2-25 | 0.6754 | 22.7 | 20.4-25.1 | 0.1931 | 20.6 | 17.9-23.7 | 0.7381 |
| *Bacillus* sp. S3 | *B. soli* DSM 15604 | 21.8 | 18.6-25.4 | 0.6660 | 23.2 | 20.9-25.7 | 0.1886 | 21 | 18.3-24.1 | 0.7290 |
| *Bacillus* sp. S3 | *B. novalis* NBRC 102450 | 21.8 | 18.5-25.4 | 0.6671 | 22.8 | 20.5-25.2 | 0.1924 | 20.9 | 18.2-24 | 0.7311 |
| *Bacillus* sp. S3 | *B. novalis* FJAT 14227 | 22 | 18.8-25.6 | 0.6612 | 23.3 | 21-25.7 | 0.1880 | 21.2 | 18.4-24.3 | 0.7249 |
| *Bacillus* sp. S3 | *B. licheniformis* ATCC 14580 | 12.9 | 10.2-16.1 | 0.9796 | 28.2 | 25.9-30.7 | 0.1524 | 13.3 | 10.9-16 | 0.9827 |
| *Bacillus* sp. S3 | *B. licheniformis* YNP1-TSU | 12.7 | 10-16 | 0.9869 | 21.2 | 18.9-23.6 | 0.2072 | 13.1 | 10.8-15.9 | 0.9896 |
| *Bacillus* sp. S3 | *B. subtilis* subsp str. W23 | 13 | 10.3-16.2 | 0.9746 | 26.1 | 23.7-28.6 | 0.1663 | 13.3 | 11-16.1 | 0.9788 |
| *Bacillus* sp. S3 | *B. subtilis* subsp str. 168 | 12.9 | 10.2-16.2 | 0.9759 | 29.5 | 27.1-32 | 0.1449 | 13.3 | 11-16.1 | 0.9794 |
| *Bacillus* sp. S3 | *B. asahii* OM18 | 13.1 | 10.4-16.4 | 0.9656 | 34 | 31.6 - 36.6 | 0.1223 | 13.5 | 11.2-16.3 | 0.9698 |
| *Bacillus* sp. S3 | *Bacillus* sp. LF1 | 14.8 | 11.9-18.2 | 0.8882 | 21.4 | 19.2-23.8 | 0.2051 | 15 | 12.5-17.8 | 0.9112 |
| *Bacillus* sp. S3 | *Bacillus* sp. OK048 | 15.7 | 12.7-19.1 | 0.8522 | 20.4 | 18.2-22.8 | 0.2154 | 15.7 | 13.2-18.6 | 0.8841 |

**Table S5.** Mobile genetic elements predicted in *Bacillus* genomes by different methods.

| Organism | Size (bp) | NO. of | | | | | |
| --- | --- | --- | --- | --- | --- | --- | --- |
|  |  | GEIs | IS | Prophage | CRISPRs | Accessory Proteins | Unique proteins |
| ***B. asahii* OM18** | |  |  |  |  |  |  |
| Chromosome | 4,826,182 | 20/5/15 | 190 | 4 | 1 | 2,414 | 1,196 |
| Plasmid | 61,312 | 0 | 42 | - | - | - | - |
| ***B. bataviensis* LMG 21833** | |  |  |  |  |  |  |
| Chromosome | 5,371,144 | 15/4/11 | 198 | 4 | 5 | 3,895 | 492 |
| ***B. cereus* SJ1** | |  |  |  |  |  |  |
| Chromosome | 5,156,400 | 7/3/4 | 105 | 1 | 1 | 3,719 | 410 |
| ***B. cucumis* strain V32-6** | |  |  |  |  |  |  |
| Chromosome | 5,707,899 | 8/2/6 | 96 | 10 | 1 | 3,813 | 545 |
| ***B. dielmoensis* FF4(T)** | |  |  |  |  |  |  |
| Chromosome | 4,556,842 | 6/2/4 | 74 | 3 | 5 | 2,939 | 519 |
| ***B. drentensis* NBRC 102427** | |  |  |  |  |  |  |
| Chromosome | 5,159,434 | 9/2/7 | 151 | 3 | 1 | 4,002 | 2 |
| ***B. drentensis* FJAT-10044** | |  |  |  |  |  |  |
| Chromosome | 5,304,541 | 22/6/16 | 8 | 3 | 7 | 4,006 | 8 |
| ***B. firmus* NBRC 15306** | |  |  |  |  |  |  |
| Chromosome | 4,421,001 | 33/20/13 | 254 | 10 | 5 | 3,415 | 15 |
| ***B. firmus* NCTC10335** | |  |  |  |  |  |  |
| Chromosome | 4,803,910 | 37/15/23 | 85 | 3 | 3 | 3,440 | 44 |
| ***B. firmus* strain 14_TX** | |  |  |  |  |  |  |
| Chromosome | 5,835,184 | 33/23/10/ | 3 | 10 | 1 | 4,143 | 615 |
| ***B. glycinifermentans* BGLY** | |  |  |  |  |  |  |
| Chromosome | 4,607,442 | 15/6/10 | 84 | 9 | 1 | 3,511 | 104 |
| ***B. glycinifermentans* SRCM103574** | |  |  |  |  |  |  |
| Chromosome | 4,744,953 | 32/17/15 | 83 | 9 | 1 | 3,564 | 139 |
| Plasmid | 65,273 | 0 | 45 | 0 | 0 | - | - |
| ***B. licheniformis* ATCC 14580** | |  |  |  |  |  |  |
| Chromosome | 4,222,597 | 16/5/11 | 70 | 4 | 0 | 3,431 | 84 |
| ***B. licheniformis* YNP1-TSU** | |  |  |  |  |  |  |
| Chromosome | 4,243,787 | 5/1/4 | 71 | 5 | 0 | 3,392 | 147 |
| Plasmid | - | - | - | - | - | - | - |
| ***B. mesonae* FJAT-13985** | |  |  |  |  |  |  |
| Chromosome | 5,804,312 | 18/10/8 | 7 | 3 | 6 | 4,226 | 174 |
| ***B. mesonae* H20-5** | |  |  |  |  |  |  |
| Chromosome | 5,839,313 | 18/9/9 | 113 | 1 | 3 | 4,249 | 181 |
| ***B. methanolicus* MGA3** | |  |  |  |  |  |  |
| Chromosome | 3,337,035 | 7/4/3 | 101 | 4 | 1 | 1,879 | 583 |
| Plasmid pBM19 | 19,174 | 0 | 0 | 0 | 0 | - | - |
| Plasmid pBM69 | 68,999 | 0 | 0 | 0 | 0 | - | - |
| ***B. niacin* DSM 2923** | |  |  |  |  |  |  |
| Chromosome | 2,199,973 | 3/1/2 | 143 | 1 | 2 | 4,338 | 513 |
| ***B. novalis* NBRC 102450** | |  |  |  |  |  |  |
| Chromosome | 5,569,548 | 23/10/13 | 97 | 3 | 15 | 4,395 | 1 |
| ***B. novalis* FJAT-14227** | |  |  |  |  |  |  |
| Chromosome | 5,667,823 | 22/6/16 | 3 | 3 | 13 | 4,402 | 10 |
| ***B. oceanisediminis* Bhandara28** | |  |  |  |  |  |  |
| Chromosome | 5,883,369 | 34/23/11 | 79 | 3 | 3 | 4,212 | 723 |
| ***B. soli* strain NBRC 102451** | |  |  |  |  |  |  |
| Chromosome | 5,464,808 | 13/3/13 | 133 | 3 | 10 | 4,274 | 11 |
| ***B. soli* strain DSM 15604** | |  |  |  |  |  |  |
| Chromosome | 5,574,519 | 14/2/12 | 4 | 5 | 9 | 4,282 | 12 |
| ***B. subtilis* subsp. spizizenii str. W23** | |  |  |  |  |  |  |
| Chromosome | 4,027,681 | 10/2/5 | 47 | 3 | 2 | 3,035 | 201 |
| ***B. subtilis* subsp. subtilis str. 168** | |  |  |  |  |  |  |
| Chromosome | 4,215,612 | 20/14/4 | 56 | 3 | 1 | 3,148 | 365 |
| ***B. thuringiensis* serovar konkukian 97-27** | |  |  |  |  |  |  |
| Chromosome | 5,314,791 | 8/1/7 | 157 | 8 | 4 | 3,936 | 383 |
| pBT9727 | 77,112 | 0 | 93 | 0 | 0 | - | - |
| ***B. thuringiensis* YBT-1518** | |  |  |  |  |  |  |
| Chromosome | 6,672,923 | 17/1/6 | 121 | 12 | 4 | 3,988 | 947 |
| pBMB0228 | 17,706 | 0 | 50 | 0 | 0 | - | - |
| pBMB0229 | 45,206 | 0 | 31 | 1 | 0 | - | - |
| pBMB0230 | 49,195 | 0 | 30 | 1 | 0 | - | - |
| pBMB0231 | 146,276 | 0 | 165 | 3 | 0 | - | - |
| pBMB0232 | 171,593 | 0 | 151 | 1 | 0 | - | - |
| pBMB0233 | 240,661 | 0 | 147 | 3 | 0 | - | - |
| ***B. vireti* LMG 21834** | |  |  |  |  |  |  |
| Chromosome | 5,283,728 | 20/9/11 | 226 | 2 | 15 | 4,182 | 3 |
| ***B. vireti* DSM 15602** | |  |  |  |  |  |  |
| Chromosome | 5,308,887 | 23/9/14 | 227 | 2 | 10 | 4,193 | 10 |
| ***B. velezensis* FZB42** | |  |  |  |  |  |  |
| Chromosome | 3,918,589 | 5/2/3 | 60 | 2 | 0 | 2,620 | 414 |
| ***Bacillus* sp. AFS006103** | |  |  |  |  |  |  |
| Chromosome | 5,175,830 | 3/1/2 | 92 | 5 | 5 | 3,678 | 344 |
| ***Bacillus* sp. OK048** | |  |  |  |  |  |  |
| Chromosome | 5,171,329 | 17/4/13 | 42 | 1 | 8 | 3,535 | 502 |
| ***Bacillus* sp. OV166** | |  |  |  |  |  |  |
| Chromosome | 7,076,838 | 27/7/20 | 2 | 1 | 4 | 4,422 | 775 |
| ***Bacillus* sp. UNC438CL73TsuS30** | |  |  |  |  |  |  |
| Chromosome | 5,605,971 | 12/4/8 | 382 | 4 | 7 | 4,007 | 324 |
| ***Bacillus* sp. UNC41MFS5** | |  |  |  |  |  |  |
| Chromosome | 6,427,278 | 15/5/10 | 59 | 4 | 6 | 4,431 | 438 |
| ***Bacillus* sp. LF1** | |  |  |  |  |  |  |
| Chromosome | 5,600,061 | 18/5/13 | 10 | 4 | 2 | 4,103 | 0 |
| ***Bacillus* sp. FJAT-18017** | |  |  |  |  |  |  |
| Chromosome | 5,265,381 | 27/22/5 | 83 | 1 | 12 | 4,104 | 0 |
| ***Bacillus* sp. FJAT-29814** | |  |  |  |  |  |  |
| Chromosome | 5,888,441 | 38/29/9 | 22 | 2 | 10 | 3,981 | 776 |
| ***Bacillus* sp. MUM 116** | |  |  |  |  |  |  |
| Chromosome | 5,720,395 | 14/4/10 | 208 | 3 | 4 | 3,848 | 444 |
| ***Bacillus* sp. X1** | |  |  |  |  |  |  |
| Chromosome | 3,422,674 | 9/3/6 | 140 | 2 | 3 | 2,138 | 281 |
| ***Bacillus* sp. 7884-1** | |  |  |  |  |  |  |
| Chromosome | 6,000,445 | 11/2/9 | 397 | 5 | 7 | 4,099 | 494 |
| ***Bacillus* sp. MRMR6** | |  |  |  |  |  |  |
| Chromosome | 5,438,352 | 11/2/9 | 96 | 3 | 4 | 3,364 | 800 |
| ***Bacillus* sp. OxB-1** | |  |  |  |  |  |  |
| Chromosome | 3,594,621 | 20/13/7 | 96 | 2 | 4 | 1,189 | 1,560 |
| ***Bacillus* sp. WN066** | |  |  |  |  |  |  |
| Chromosome | 6,213,578 | 18/6/12 | 100 | 2 | 7 | 4,383 | 339 |
| ***Bacillus* sp. S3** | |  |  |  |  |  |  |
| Chromosome | 5,436,472 | 12/4/8 | 114 | 5 | 7 | 3,893 | 385 |
| Plasmid | 241,339 | 3/3 | 47 | 0 | 0 | - | - |

"-": unpublished or unfinished. GEIs, IslandPath-DIMOB/SIGI-HMM/IslandPick.

**Table S6.** Primers used in RT-qPCR

| Genes | Sequence (Forward primer/Reverse primer) | Annealing temperature (℃) | Length (bp) |
| --- | --- | --- | --- |
| *aioB* | 5’- GGTTGATTTCGCTTTCCCTG-3’ | 53.4 | 155 |
|  | 5’- CGTGATGACAAGGGCAAAGC-3’ | 55.4 |  |
| *arsB_1* | 5’- GCCACCATTCCTACTCA -3’ | 52.2 | 182 |
|  | 5’- CGCTACAAGTGATACGG -3’ | 52.2 |  |
| *arsB_2* | 5’- TTGCTATCCCAGTTTCAC -3’ | 48 | 189 |
|  | 5’- CAATCAGCCAGTCCGTTA -3’ | 51.8 |  |
| *arsB_3* | 5’- TGAAGCGATTCGGGATGA-3’ | 57.5 | 130 |
|  | 5’- GAAGACGGCAACGACACC-3’ | 56.4 |  |
| *arsC* | 5’- TAGGTGATGAGTGGGAAGTG-3’ | 55.4 | 251 |
|  | 5’- ACCTTCTGCTTTCGCTGGAT-3’ | 55.4 |  |
| *psts_1* | 5’- CGTTCTTGTAAACCGTCCTG -3’ | 55.4 | 150 |
|  | 5’- TAACCAATCGCACCTTCTG -3’ | 53 |  |
| *16s* | 5’-AGAGTTTGATCCTGGCTCAG-3’ | 53.4 | 191 |
|  | 5’-GGTTACCTTGTTACGACTT-3’ | 55.6 |  |

**Table S7.** The quality control of clean data.

| Metrics | Raw Reads | Filtered Reads |
| --- | --- | --- |
| Polymerase Read Bases | 1,055,530,596 | 874,728,983 |
| Polymerase Reads | 150,292 | 88,313 |
| Polymerase Read N50 | 13,074 | 13,621 |
| Polymerase Read Length | 7,023 | 9,904 |
| Polymerase Read Quality | 0.61 | 0.846 |

**Table S8.** The statistical information and of clean data.

| Job Metric | Value |
| --- | --- |
| Polished Contigs | 2 |
| Adapter Dimers (0-10bp) | 0.01% |
| Short Inserts (11-100bp) | 0.00% |
| Number of Bases | 874,728,983 |
| Number of Reads | 88,313 |
| N50 Read Length | 13,621 |
| Mean Read Length | 9,904 |
| Mean Read Score | 0.85 |
| Mapped Reads | 79,457 |
| Mapped Read Length of Insert | 7,429 |
| Average Reference Length | 2,858,339 |
| Average Reference Bases Called | 100.00% |
| Average Reference Consensus Concordance | 99.99% |
| Average Reference Coverage | 110.81 |
| Coverage level | 874.7/5.4 |

**Table S9.** The statistical information of genome sequencing and assembling procedures.

| Class | Value |
| --- | --- |
| coverage level (x) | 874.7/5.4 |
| Contig N50 (bp) | 163,277 |
| kmer length (bp) | 127 |
| Coverage | 99.99% |
